# Homelessness prediction models in high-income countries: a scoping review

**DOI:** 10.1186/s12889-025-24855-x

**Published:** 2025-11-17

**Authors:** Luis Antonio Stängl, Evelina Baniunaite, Daniel Fürstenau, Stefanie Schreiter, Katherine A. Koh, Chisato Ito, Derin Marbin

**Affiliations:** 1 Charite-Universitätsmedizin Berlin, corporate member of Freie Universität Berlin, Humboldt Universität zu Berlin, Berlin, Germany; 2 School of Business & Economics, Freie Universität Berlin, Berlin, Germany; 3 Department of Psychiatry and Neurosciences, Charité - Universitätsmedizin Berlin, corporate member of Freie Universität Berlin, Humboldt-Universität zu Berlin, Berlin Institute of Health, Berlin, Germany; 4 Institute of Public Health, Charité - Universitätsmedizin Berlin, Freie Universität Berlin and Humboldt Universität zu Berlin, Berlin, Germany; 5 Department of Psychiatry, Massachusetts General Hospital, Boston, MA US; 6 Boston Health Care for the Homeless Program, Boston, MA US; 7 Department of Psychiatry of University, Hospital Charité in St. Hedwig Hospital Berlin, Berlin, Germany; 8 Institute of Medical Informatics, Charite- Universitätsmedizin Berlin, Universität Berlin and Humboldt Universität zu Berlin, Berlin, Germany

**Keywords:** Homeless, Prediction, Prediction model, Prevention, Scoping review

## Abstract

**Objective:**

This scoping review aims to provide an overview of prediction models for future homelessness in high-income countries.

**Background:**

Several risk prediction models for homelessness have been developed and are being used in practice. However, no comprehensive review has captured the full scope of these models in regard to their target populations, data and variables used, type of model, and model validation.

**Methods:**

We searched MEDLINE, Web of Science, the Cochrane Library, and Bielefeld BASE without language or time restraints. Following the JBI guidelines, screening was performed by two independent reviewers; the data were extracted by one, of which 20% was checked by a second one. Studies that reported on the development or validation of prediction models for becoming homeless in high-income countries, and whose study population included individuals residing in these countries who were not homeless at the time of recruitment, were included.

**Results:**

Our search resulted in 9,371 deduplicated records across databases. 15 studies met the inclusion criteria, of which 14 were model development studies and one was a validation study. 13 studies (87%) were conducted in the US, six of them in New York City (NYC). One study was conducted in Canada and one in Australia. Regarding the target population, three studies developed models for veterans and six studies targeted welfare applicants or recipients. One study focused on both youth emerging from public assistance and unemployed workers. Three studies developed models for the general population, while two were conducted in emergency departments. Of the 15 studies, 14 used traditional regression, seven employed other machine learning algorithms and six used both methods. The most common predictor types were: demographics, age, previous experiences of homelessness, human capital such as employment status or total debt and clinical variables such as physical or mental health status. Three studies combined geographical-level and individual-level data. In total, 25 models were identified, two of which were externally validated.

**Conclusions:**

We found a broad spectrum of heterogeneity of models and population studies, an increase in model development over time, and limited use of calibration metrics. Prediction models for future homelessness have the potential to improve risk targeting and the effectiveness of preventive programs. As only two models were externally validated, we recommend that future research focuses on model evaluation.

**Supplementary Information:**

The online version contains supplementary material available at 10.1186/s12889-025-24855-x.

## Introduction

The number of people experiencing homelessness remains high and is increasing in most high-income countries despite their social security systems and overall wealth [1, 2].

Homelessness is a critical and complex state of social exclusion influenced by multiple factors on structural, political, community, interpersonal and individual levels. Structural factors, for example, involve income inequality, lack of affordable housing, employment opportunities and welfare systems [1, 3, 4]. Individual factors include substance use disorders, other mental health issues, adverse childhood experiences, individual poverty and criminal justice involvement [1]. The consequences of homelessness extend beyond the absence of shelter, significantly impacting physical and mental health as well as the quality and length of life of those affected [2].

The European Union has attempted to standardize the definition of homelessness with the introduction of the European Typology of Homelessness and Housing Exclusion (ETHOS) in 2006 [5]. ETHOS provide four main categories of housing exclusion and homelessness: sleeping outside (rooflessness), staying at emergency institutions (houselessness), living in insecure housing (e.g., on friends couches or being susceptible to domestic violence), and living in inadequate housing (e.g., caravans or extreme overcrowding) [5, 6]. The first two categories are generally considered as homelessness, while the latter two are considered as housing exclusion [5]. Consistent definitions of homelessness across countries and studies are currently not applied, making the comparison of studies difficult [1, 2].

Researchers often use their specific country definitions, or create their own and refer to ETHOS when trying to compare their definitions or numbers. Since many studies originate in the US, the definition of the US Department of Housing and Urban Development (HUD) is frequently referred to. The HUD definition differentiates literal homelessness: Individuals who lack a fixed, regular, and adequate nighttime residence—and at-risk categories: people who are on the verge of losing their housing or are living in temporary, unstable, or inadequate arrangements [7].

Preventing people from entering homelessness can protect them from downward spirals, including traumatic experiences on the street or in shelters, experiences of discrimination and developing or worsening of mental and physical health problems [8, 9], which are in part due to increased difficulty in engaging in treatment while homeless [10]. From a community or societal perspective, prevention services such as housing subsidies have been shown to be more cost-efficient than emergency shelters and reintegration programs [11]. Furthermore, a study suggests that when moving chronically homeless people into housing it often remains difficult to improve morbidity and mortality outcomes, thus underscoring the urgency of preventing people from becoming homeless in the first place [12].

To increase the efficiency of prevention programs, it is important to accurately target populations who could otherwise become homeless [13]. Risk assessment should be evidence based, using validated models [8, 14]. In many US cities, risk targeting is often either based on a single factor model of eviction, in which programs focus on preventing people from being evicted [8]. However, the effectiveness of eviction as a predictor for future homelessness and the accuracy of these programs in targeting those truly at risk lacks an evidence basis. In fact, one study suggests that the majority of those who are evicted do not become homeless [15]. Similarly, individual assessments by social workers, another commonly used method, also lack an evidence base for their predictive ability, as it has not been evaluated [8, 9]. Prediction models could provide a sophisticated additional method of risk assessment to relying only on eviction status or the subjective judgment of social workers [8, 9, 16].

In fields such as biomedicine, prediction models for future health outcomes, such as cardiovascular diseases, are well established and used in a variety of contexts and settings [14, 16, 17]. An example would be the Framingham risk score for cardiovascular diseases [18]. These models can be created using either regression methods, such as logistic regression and Cox regression, or by machine learning (ML) methods, such as random forests [14]. In the context of homelessness prevention services, several studies have successfully developed and implemented prediction models [8, 9, 16, 17].

While prediction models have been developed and used in practice [9, 13], there is no comprehensive review that captures the full scope of these models in regard to their target populations, data and variables used and type of model. A preliminary search conducted on April 1, 2024, revealed only one systematic review, which focused on ‘individual-level predictors’ and sought to identify causal factors of homelessness rather than risk prediction models [19].

The objective of this review was to identify the current scope of prediction models that aim to predict the risk of individuals—who are not currently homeless or live in inadequate housing—of becoming homeless in high-income countries. Specifically, this review sought to identify the following: the predominant methods used in the model development; training data used; potential use cases; settings and target populations; individual- and geographical-level predictors selected in the models; the observation time; the definition and measurement of the primary outcome; model validation; and model performance measures used.

The study focused on high-income countries due to the relative comparability of their social security systems [20], as well as the contrast between their overall wealth and the extreme form of poverty and social exclusion that homelessness represents [21, 22]. Low- or middle-income countries might differ significantly in their welfare systems, housing and labor markets and thus the applicability of prediction models developed in high-income settings may be limited.

## Methods

This scoping review was conducted in accordance with the guidelines of the Johanna Briggs Institute (JBI) for scoping reviews [23] and is reported following the Preferred Reporting Items for Systematic reviews and Meta-Analyses extension for Scoping Reviews (PRISMA-ScR) [24]. A protocol was published on the Open Science Framework (OSF) on May 17, 2024.

### Eligibility criteria

We defined eligibility criteria according to The JBI Population Concept Context (PCC) framework [23]:

- Population: Individuals who are currently not experiencing homelessness, including those living in the third or fourth ETHOS category (insecure housing and inadequate housing) [5].
- Concept: Prediction models for becoming homeless as an outcome. A prediction model can be developed utilizing either regression methods, such as logistic regression, or by employing ML algorithms such as random forests [14].
- Context: Homelessness in high-income countries (>13845 Atlas Gross National Income (GNI) per Capita) [25]. Homelessness was a binary outcome to be predicted, defined as sleeping and living outside, or sleeping at emergency shelters according to the ETHOS definitions [5].

The inclusion and exclusion criteria were defined in accordance with the CHecklist for critical Appraisal and data extraction for systematic Reviews of prediction Modelling Studies (CHARMS) [26]. Included were studies that aimed to develop prediction models for individuals entering homelessness. Both development and validation studies were eligible. Excluded were studies that focused on models for people currently experiencing homelessness [27], and that predicted homelessness prevalence [28], as well as single predictor discovery studies [19]. A complete list of inclusion and exclusion criteria can be found in Appendix A.2. No time or language constraints were applied. Articles in languages other than English and German were translated via DeepL [29]. However, the search string was developed in English and not translated to other languages. Gray literature such as master’s theses or dissertations were included. Non-academic articles, reports, or websites published by governmental or other institutions were excluded if the methodology was not reported.

### Search strategy

We used a previously developed and validated search filter for prognostic prediction modeling studies [30] in combination with a search string for homelessness, which we developed with the help of a librarian. While homelessness is a state, not an illness, this review is based on biomedical literature, where the term “prognostic” is used to distinguish models that are intended to predict future homelessness from “diagnostic” models, which aim to detect current homelessness. The full search string can be found in Appendix A.1., along with translations for the different databases. On April 24, 2024, we searched the following databases: MEDLINE via Ovid, Web of Science, the Cochrane Library and Bielefeld Academic Search Engine (BASE) (*base-bielefeld.net*), a database for gray literature. Additionally, on May 30, 2024, we used the citation-chaser website to find other relevant studies through backwards and forwards reference screening [31].

### Selection process

**Fig. 1:**
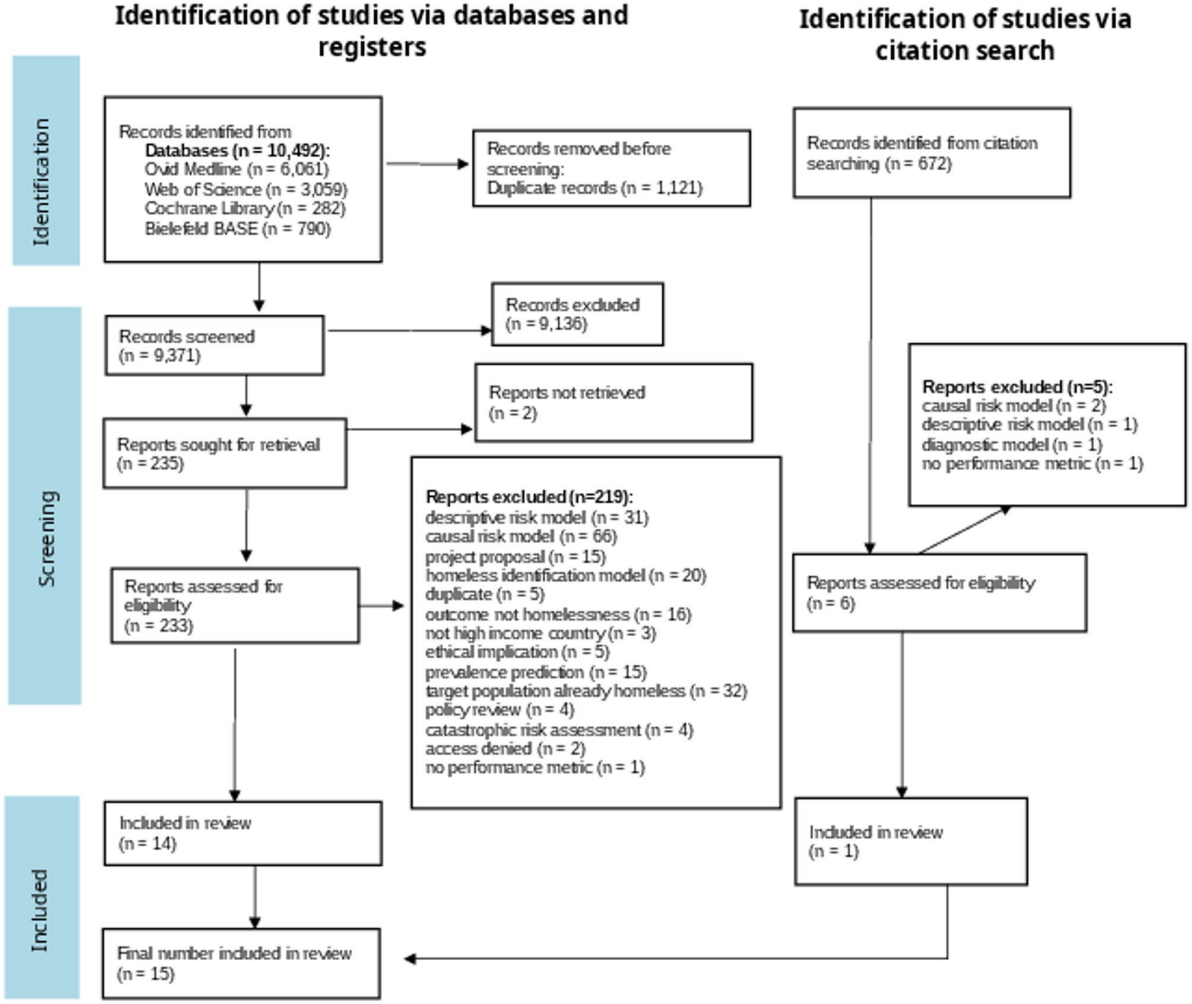
Prisma Flow diagram

Identified articles were exported to Zotero (v6.0.35.) [32]. The Systematic Review-Accelerator website [33] was used for identifying duplicates and for the screening process. Two independent reviewers (LAS and EB) performed both the title and abstract screening and the full-text screening. A calibration process was initiated beforehand to guarantee agreement between the two reviewers. Disputes of screened texts were solved by consensus between LAS and EB. Information on the reasons for exclusions is reported in a flow chart according to PRISMA [34] in Fig. 1.

### Data extraction

The extraction form was developed based on the CHARMS checklist [26] and a pilot testing was conducted. During the pilot testing, the extraction form was first tested on three studies and then revised to better capture the variables of interest. The following variables were extracted: study and model characteristics, such as study population and size, model development methods, model performance measures, model evaluation measures, model results and model interpretation. The full extraction form can be found in Appendix A.3. The data extraction form was created as a Zotero template and filled out in Zotero (v6.0.35) [32]. The data extraction was performed by LAS using the developed extraction form. EB checked a random sample of 20% of the extracted data for correctness, following the JBI guidelines [23]. After finishing each extraction form, the data were transferred to LibreOffice Calc (v24.2.4.) [35].

### Data synthesis

The results were summarized narratively according to the JBI guidelines [23] and the CHARMS Checklist, excluding the risk of bias assessment [26]. The risk of bias assessment is the quality measure used for systematic reviews of prediction models. This assessment was out of scope of this scoping review as there is no quality assessment of the included studies done in a scoping review [23]. Descriptive statistics were used to summarize the extracted data with R (v4.4.0) [36], through RStudio (v2.04.24) [37] and LibreOffice Calc [35]. The tables are created following the CHARMS guidelines, and the figures were based on JBI and the recommendations from Pollock et al. [38].

### Protocol deviations

The data were not, as indicated in the protocol, extracted by two reviewers, but instead extracted by LAS, and 20% of the data were checked by EB. This was due to time and resource constraints. In addition, we limited the extraction of the model performance measures only to the area under the receiver operator characteristic curve (AUC) values, as evaluation of the model performances was outside the aim of this scoping review. Additionally, only the CHARMS checklist was used for our extracted variables and the Transparent reporting of multivariable prediction models for individual prognosis or diagnosis: checklist for systematic reviews and meta-analyses (TRIPOD-SRMA) was left out, as it was not applicable.

## Results

### Search results

The search results are illustrated in Fig. 1. The search resulted in 10,492 hits overall on April 24, 2024. A total of 6,061 records were identified in Ovid MEDLINE, 3,059 in Web of Science, 282 in the Cochrane Library, and 790 in Bielefeld BASE. After removing 1,121 duplicate records, 9,371 records were screened based on titles and abstracts. Following this screening process, 9,136 were excluded, and 235 selected for retrieval. A full-text screening was performed on 233 studies, as two studies were not accessible after contacting the authors. Of these, 219 studies were excluded, primarily because they developed descriptive [39] (*n* = 32) or causal risk models (*n* = 66), diagnostic models (*n* = 20), or focused on populations that were already experiencing homelessness (*n* = 32). Descriptive models often described profiles of youth experiencing homelessness [39]. Ultimately, 14 studies were included from the lrature search. Additionally, the citation chaser was used to conduct forward and backward citation searches. Six full texts were retrieved, and one study was included, resulting in a total of 15 studies included in this scoping review. The full list of the included references, as well as well as those excluded during the full text screening, and their corresponding exclusion classifications can be found in Appendix A.7.

### Study overview

**Fig. 2:**
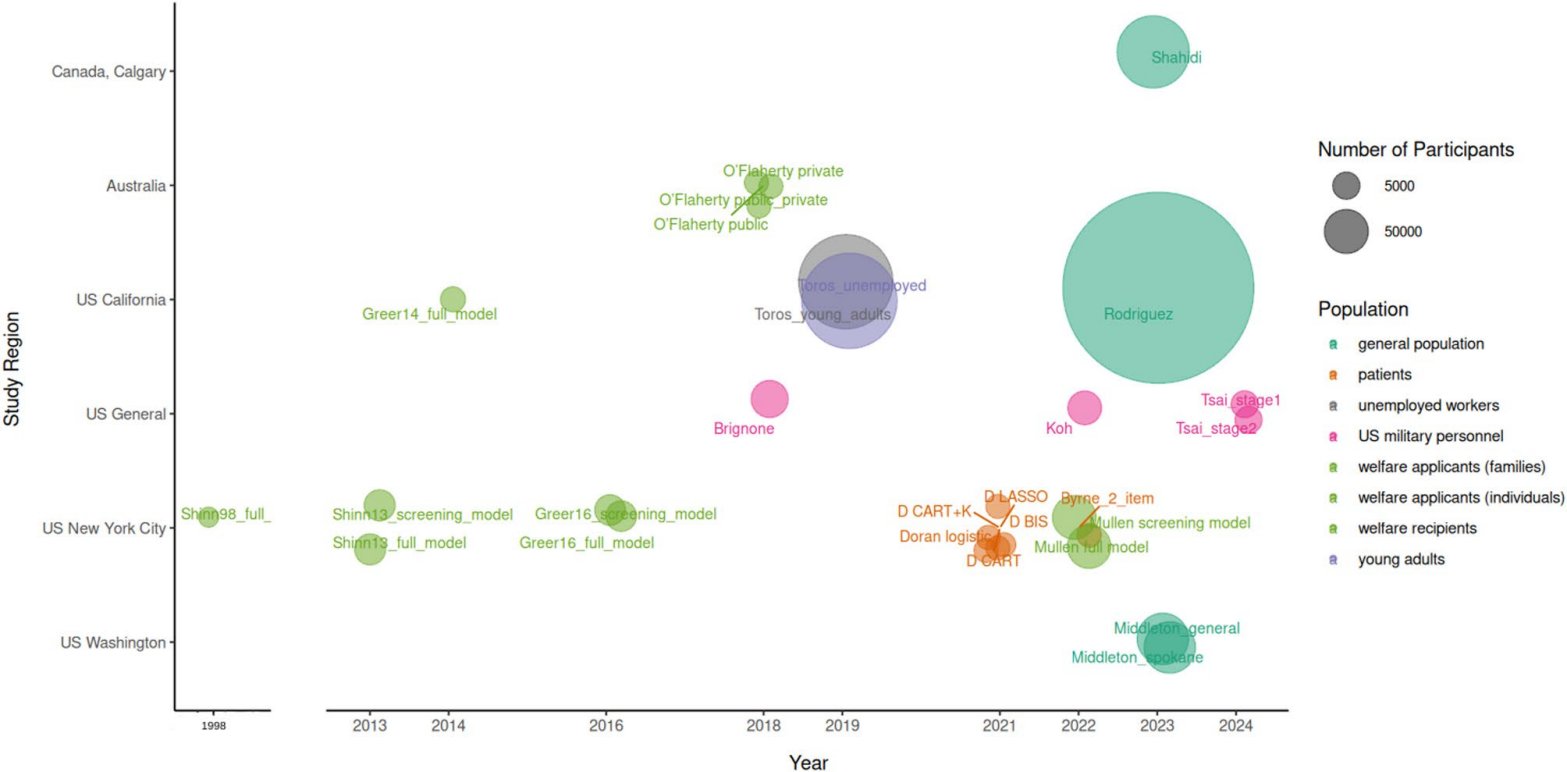
Study and model overview. Each bubble represents a model developed in a study. Text: model name, text color: study population, size of bubble: study population size. Us General refers to data from military personnel across the US. The gap in the x-achsis is a jump from 1998 to 2013, where no models were developed

Table 1 and Fig. 2 provide an overview of the study and model characteristics. The study setting and year, data source, study region, number of models, study population, and modeling methods are shown in Table 1. The model characteristics are shown in Table 2 and Fig. 2.

**Table 1:** Study characteristics

Study + Year	Data Source	Study region	*N*° of models	Population	Modeling method
Brignone et al., 2018 [40]	Veteran health administration	US	1	US veterans	Random forest (machine learning)
Byrne et al., 2022 [41]	Patient surveys and administrative data	New York City, New York, US	1	Patients in public hospital emergency department	Logistic regression
Doran et al., 2021 [42]	Patient surveys and administrative data	New York City, New York, US	4	Patients in public hospital emergency department	Logistic regression, CART (machine learning), LASSO regression
Greer, 2014 [43]	Homelessness prevention program	Alameda County, California, US	1	Welfare applicants (individuals)	Cox proportional hazards regression
Greer et al., 2016 [17]	Homelessness prevention program	New York City, New York, US	2	Welfare applicants (individuals)	Cox proportional hazards regression
Koh et al., 2022 [44]	Army/Department of Defense administrative data; personnel surveys; geospatial data	US	1	US army soldiers no longer on active duty	0-fold cross-validation super learner (machine learning), LASSO regression
Middleton et al.,2023 [45]	Utility data of electricity and gas providers	Spokane City, Washington, US	2	Residents of Spokane City	Binary logistic regression
Mullen et al., 2022 [9]	Homelessness prevention program	New York City, New York, US	2	Welfare applicants (families)	Cox regression
O’Flaherty et al., 2018 [46]	Australian welfare program	Australia	1	Welfare recipients	Logistic regression
Rodriguez et al., 2023 [47]	Electronic health records	Northern California, US	1	Insured general population	Logistic regression, random forest(machine learning)
Shahidi et al., 2023 [48]	Administrative health care data	Calgary, Canada	1	Insured general population	Linear regression, random forest, XGBoost (machine learning)
Shinn et al., 1998 [49]	NYC welfare program and administrative data	New York City, New York, US	1	Welfare applicants and receivers (families)	Logistic regression
Shinn et al., 2013 [8]	Homelessness prevention program	New York City, New York, US	2	Welfare applicants (families)	Cox regression
Toros et al., 2019 [50]	Administrative records	California, US	2	Young adults, unemployed workers	Logistic regression, Random forest (machine learning)
Tsai et al., 2024 [16]	Army//Department of Defense administrative data; survey data; geospatial variables	US	2	US army transitioning service members	Random forest (machine learning), LASSO regression

*LASSO* Least Absolute Shrinkage and Selection Operator, *CART* Classification and Regression Trees, *XGBoost* Extreme Gradient Boosting

The study by Shinn et al. in 1998 [49] was the oldest study that met the inclusion criteria and was conducted in NYC, targeting families requesting shelter as well as families receiving welfare. The 2013 study by Shinn et al. [8] was conducted in a similar setting and is referenced by nine other included studies as one of the original homelessness prediction modeling studies [9, 17, 42–47, 50]. The number of models developed and the study population size increased over time (Fig. 2). As depicted in Table 1 and Fig. 2, 13 of 15 studies were conducted in the United States, one in Canada [48], one in Australia [46], and none in Europe, Asia or South America. Six of the 15 included studies were conducted in one city: NYC [8, 9, 17, 41, 42, 49]. Three studies (four models) were conducted in California [43, 47, 50]. One study was conducted in Spokane City, Washington [45] and three studies used data from across the entire US [16, 40, 44].

### Study purposes

Of the 15 included studies, 14 developed a new model. The study from Mullen et al. [9] externally validated and updated Shinn et al.’s [8] full model as well as their screening model. A screening model is a shorter model that can be used with a point scoring system without a computer. Greer [43] and Greer et al. [17] developed their models based on Shinn et al.’s [8] model, but performed no external validation and no model update. Models can be updated if a validation study finds better predictive performance through adjusting an existing model.

Two studies differed in their study purposes from the others, whose main aim was to develop prediction models: O’Flaherty et al. [46] and Shahidi et al. [48] both developed their models to answer research questions alongside their model development. O’Flaherty et al. [46] developed three models in Australia with welfare recipients, showing that models that incorporate “private” information—information that is not publicly available, such as self-assessed homelessness risk, total debt, or social networks—have a higher predictive performance compared to models that do not use this kind of data. Byrne et al. [41] and Mullen et al. [9] tried to include such “private” information in their models. Shahidi et al. [48] used administrative health care data from Canada to demonstrate that flexible cohorts enhance the predictive ability of models about future homelessness. Flexible cohorts are cohorts that utilize information about individuals spanning all the available time frames that are present in the data for these individuals, whereas fixed cohorts utilize fixed time frames for the whole cohort. If data was present for specific individuals in a cohort from 2015 to 2020 but for the whole cohort only from 2015 to 2018, using the available longer time frame for parts of the cohort would be a flexible cohort. Using only the data for all individuals from 2015 to 2018 would be a fixed cohort.

### Study population size

The bubble size in Fig. 2 corresponds to the size of the study population. The smallest study, conducted by Shinn et al. [49] in 1998 had a sample size of 566 participants, while the largest, conducted by Rodriguez et al. [47], had access to data from 2.5 million individuals. Since 2018, more studies have used larger datasets [45, 47, 48, 50].

### Data collection

Five studies involved primary data and secondary data collection [8, 16, 17, 41, 42, 49], while eight used exclusively secondary data [40, 43–48, 50]. Secondary data was veteran and military administrative data [16, 40, 44], communal administrative data [8, 9, 17, 41–43, 45, 50], health insurance administrative data [47, 48] and large-scale survey studies [16, 40, 44, 46].

### Study populations

Figure 1 and Table 1 show the study populations of the studies. The color in Fig. 1 corresponds to the study population. Welfare applicants and recipients were targeted by six studies and nine models [8, 9, 17, 43, 46, 49].

Shinn et al. 1998 [49] studied two cohorts of families, one requesting shelter and one receiving welfare benefits. Shinn et al. 2013 [8] targeted families in NYC who were applying for homelessness prevention services. The studies by Greer [43], Greer et al. [17], and Mullen et al. [9] were conducted in the same context. In Greer et al. [17], the full model from Shinn et al. [8] was redeveloped for single individuals from the general population applying for homelessness prevention services. In Greer [43], Shinn et al.’s [8] full model was redeveloped for single individuals in a different county, Alameda County, in Southern California. Mullen et al. [9] conducted an external validation of Shinn et al.’s [8] models and updated them. O’Flaherty et al. [46] developed their models in Australia targeting individuals who were already receiving welfare benefits. Four models were developed for the general population [45, 47, 48], and another six were developed for emergency department patients [41, 42]. Four models focused on US military personnel or veterans [16, 40, 44], one on unemployed workers [50], and one on young adults emerging out of public assistance [50]. Except for Toros et al.’s young adults model, no model focused on a specific age group.

As depicted in Tables 1 and 2, neither gender nor sex were used to specify the target population. However, as shown in Table 2, the homelessness prevention studies that focused on families had a study population that was more than 90% female [8, 9, 49], while studies focusing on military personnel or US veterans reported that females comprised between 8% and 16% [16, 40, 44]. For the five other studies, where gender (no differentiation was made between sex and gender) was reported, the gender distribution was relatively balanced with an average of 45.4% female and a standard deviation of 5.1 (own calculations based on: [17, 41, 42, 47, 48]). Other demographic characteristics, such as race and ethnicity, were not used to specify target populations.

**Table 2:** Model characteristics

Study	Model description (Specifics of the developed model, either goal or method if several models were developed)	Female %	Time to Outcome (months)	*N*° Partici-pants	*N*° Predictors	Type of Final Predictor included in the model (original categories)
Brignone et al. [40]	Prediction of future homelessness for veterans	7.8	1–12	25,510	na	Demographic, military service status, health status, and healthcare utilization characteristics
Byrne et al. [41]	Two item screening tool for emergency department patients	49	2–12	1919	2	Self-assessed current and future risk
Doran et al. [42]	Logistic regression model	47.5	6	1993	12	Human capital, homelessness history, self assessed future risk, clinical, housing conditions, demographics
Doran et al. [42]	CART model	47.5	6	1993	9	Self-assessed risk, human capital, housing conditions, clinical, criminal history, homelessness history, self assessed risk, housing conditions clinical
Doran et al. [42]	CART model with k-fold cross validation	47.5	6	1993	3	Human capital, homelessness history, demographics
Doran et al. [42]	Logistic model with Bayesian Information Criterion	47.5	6	1993	1	Homelessness history
Doran et al. [42]	Full 10-fold cross-validation with selection of variables using LASSO	47.5	6	1993	6	Criminal history, homelessness history, self assessed risk, homelessness history
Greer [43]	Prediction of future homelessness for individuals in Alameda	na	Unclear	2761	9	Demographics, human capital, housing conditions, shelter history
Greer et al. [17]	Prediction of future homelessness for individuals in New York City	41.8	12–96	10,220	37	Demographics, human capital, housing conditions, disability, interpersonal discord, childhood experiences, and shelter history.
Greer et al. [17]	Questionnaire version of the model	41.8	12–96	10,220	7	Demographics, human capital, housing conditions, disability, shelter history.
Koh et al. [44]	Prediction of future homelessness in US soldiers no longer on active duty	na	12	16,589	26	Mental illness, adverse childhood experiences, lifetime traumas, geospatial variables
Middleton et al. [45]	Prediction of future homelessness based on utility payment data. General model	na	12	86,317	5	Number of persons at household, money owed, pay plan deviations
Middleton et al. [45]	Model for Spokane city, uses more specific data	na	12	86,318	14	Specific utilities not paid
Mullen et al. [9]	External validation of Shinn et al.’s [8]model	90.5	36	48,450	47	Sociodemographics, child-related, return to residence, housing items, childhood adversity
Mullen et al. [9]	External validation of Shinn et al.’s [8] screening model	90.5	36	48,450	47	Sociodemographics, child-related, return to residence, housing items, childhood adversity
O’Flaherty et al. [46]	The use of private, not publicly available information, adds predictive values to homelessness prediction models (combined model)	na	3–12	1919	39	Demographics, human capital, childhood experiences, clinical, housing conditions
O’Flaherty et al. [46]	Using only public information	na	3–12	1919	39	Housing conditions, homelessness history, human capital, clinical, interpersonal history, demographics, childhood experiences, criminal history
O’Flaherty et al. [46]	Using only private information	na	3–12	1919	39	Housing conditions, homelessness history, human capital, clinical, interpersonal history, criminal history
Rodriguez et al. [47]	Prediction model using electronic health records. To be used in inpatient, outpatient, or emergency department settings.	52.8	12–24	2,543,503	26	Demographics, clinical, geographical-level
Shahidi et al. [48]	Comparison of predictive performance of homelessness prediction models using either a fixed cohort or a flexible cohort.	36.2	24	237,602	28	Gender, age, emergency department information, clinic history visits, comorbidities
Shinn et al. [49]	Prediction of future shelter entry for families receiving welfare	95	60	563	21	Demographics, persistent poverty, disorder, social ties, housing
Shinn et al. [8]	Prediction of future shelter entry for families applying for homelessness prevention services. Shows that prediction models can make such programs more efficient.	91.7	36	11,105	41	Demographics, human capital, housing conditions, disability and criminal justice, interpersonal discord, childhood experiences, shelter history self-reported, shelter history administrative data
Shinn et al. [8]	Questionnaire version of the model	92.7	36	11,106	15	Demographics, human capital, housing conditions, disability and criminal justice history, interpersonal discord, childhood experiences, shelter history self-reported, shelter history administrative data
Toros et al. [50]	Prediction of future homelessness for youth transitioning out of social services	na	36	479,111	22	Demographics, employment, homelessness history, health and behavioral health, criminal justice status, social services use, foster care history.
Toros et al. [50]	Prediction of future homelessness for unemployed workers	na	36	494,584	43	Demographics, employment, homelessness history, health and behavioral health, criminal justice, social services
Tsai et al. [16]	Identifies transitioning veterans at high risk of homelessness within 12 months of leaving the military. Uses individual and geographical data	16	12	4790	10	Unemployment rate in county, widow rate, age rate, unemployed veteran rate, discharge status, child food insecurity rate in county, junior enlisted rank
Tsai et al. [16]	To be used on individuals identified by the first model, is based on existing administrative data and a 10 question self-assessment	16	12	4790	10	Dependents, religiosity, physical health, mental health, suicidality

human capital: a category of economic and social attributes, originally used by Shinn et al. ‘s 2013 study. *na* not available data, not reported by the study

### Outcome definitions

All studies used homelessness as a binary variable. The six NYC studies used shelter entry to define homeless status, as NYC has the ‘right to shelter,’ with over 90% of people experiencing homelessness being sheltered, providing a usable proxy [8, 17, 41, 42, 49]. Other classification systems of homelessness varied and are compared in Table 3. The ETHOS [5] categories of ‘rooflessness’ and ‘houselessness’ are similar to the definition of ‘literal homelessness’ by the US Department for Housing and Urban Development (HUD) [7], the American version of the International Classification of Diseases-Version 10 (ICD-10) [51] definition, as well as the definition of ‘primary and secondary homelessness’ by Chamberlain and Mackenzie [52]. Middleton et al. [45] used the HUD definition [7]; O’Flaherty et al. [46] utilized both the HUD definition and ‘cultural homelessness’, which was defined by Chamberlain and Mackenzie and was used, among others, by the Australian Bureau of Statistics [53]. Toros et al. [50] and Tsai et al. [16] modified the HUD definition. Greer [43] combined the HUD definition with the ETHOS categories. Rodriguez et al. [47], Shahidi et al. [48] and Brignone et al. [40] used the ICD-10 codes Z590, Z591 and the ICD-9 codes V600 and V601 for homelessness [51, 54]. In Appendix A: Supplemental Table 1, the definitions used by each study are summarized. Greer [43] and O’Flaherty [46] used their models to predict housing exclusion alongside homelessness.

**Table 3:** Comparison of homelessness definitions

	ETHOS [5]	HUD [7]	ICD-10, Z59.0 (American Version) [51]	Cultural Definition [47]
Homelessness	1) Rooflessness (without a shelter of any kind, sleeping rough)	Category 1: Literally homeless: Individual or family who lacks a fixed, regular, and adequate nighttime residence, meaning: Has a primary nighttime residence that is a public or private place not meant for human habitation; or Is living in a publicly or privately operated shelter designated to provide temporary living arrangements	Persons lacking permanent or reliable shelter, variously due to poverty, lack of affordable housing, mental illness, substance abuse, juvenile alienation, or other factors.	Primary homelessness: people without conventional accommodation (living on the streets, in deserted buildings, improvised dwellings, under bridges, in parks, etc.)
	2) Houselessness (with a place to sleep but temporary in institutions or shelter)			
				Secondary homelessness: people moving between various forms of temporary shelter including friends, emergency accommodation, youth refuges, hostels and boarding houses
Housing exclusion	3) Living in insecure housing (threatened with severe exclusion due to insecure tenancies, eviction, domestic violence)	Category 2: Imminent Risk of Homelessness: An individual or family who will imminently lose their primary nighttime residence		
		Category 4: Fleeing/Attempting to Flee Domestic Violence		
	4) Living in inadequate housing (in caravans on illegal campsites, in unfit housing, in extreme overcrowding).	Category 3: Homeless Under Other Federal Statutes: Unaccompanied youth under 25 years of age, or families with Category 3 children and youth, Have not had a lease, ownership interest in permanent housing during the 60 days prior to the homeless assistance application; Have experienced persistent instability as measured by two moves or more during in the preceding 60 days;		Tertiary homelessness: people living in single rooms in private boarding houses without their own bathroom, kitchen or security of tenure

The first row of Table 3 shows definitions of what is generally considered homelessness, while the second and third rows show definitions for populations experiencing housing exclusion

*ETHOS* European Typology on Homelessness and Housing Exclusion, *HUD* US Department of Housing and Urban Development, *ICD* International Classification of Diseases

### Overview of predictive modeling approaches

As depicted in Tables 1 and 13 out of 15 studies used either Cox, logistic, or least absolute shrinkage and selection operator (LASSO) regression, and seven studies utilized more complex machine learning algorithms [16, 40, 42, 44, 47, 48, 50], five of which used random forest [16, 40, 47, 48, 50].

Only Brignone et al. [40] did not use any regression methods. In five studies, the authors utilized both regression methods and more complex machine learning algorithms [16, 42, 44, 47, 50]. In three of these studies, the authors argued they wanted the models to be transparent and understandable, stating that models developed by more complex machine learning algorithms are more of a “black box,” making them harder to comprehend and more difficult to create a scoring system from [16, 47, 50]. The prediction time frame spanned from one to 96 months (eight years).

Except for Doran et al. [42], who developed five models using different data regression methods and machine learning algorithms, and O’Flaherty et al. [46], who developed three models, all studies developed one or two models.

Of the five studies that developed two models [8, 16, 17, 45, 50], two studies [8, 17] created a full model and a screening model. The screening models were shorter and used point scoring systems, which were intended to be used by social workers. The full models used the developed coefficients, the screening models transformed the coefficients to points, so they were easier to use and calculate without a statistical program.

Doran et al. [42] reported developing a large number (*n* = 230) of screening tools from their full models, presenting 21 exemplary ones in their publication. They created a wide range of screening models and intended to gather input from stakeholders to determine their usefulness.

O’Flaherty et al. [46] developed three models, one using private information, one using public information and one using both. Tsai et al. [16] created one model which used exclusively geographical-level data for a primary risk calculation and a second model which used individual-level data. Middleton et al. [45] created one model for Spokane, Washington, from where they obtained the data for the model development, and a shorter, general model for use in other cities. Toros et al. [50] created two models for differing target populations.

## Discussion

This review provides a comprehensive overview of the scope of models predicting future homelessness in high-income countries. A total of 15 studies met our inclusion criteria, of which one was a validation study [9] and 14 were model development studies. One study was conducted in Canada [48] and one in Australia [46]. The other 13 studies were conducted in the US, of which six were conducted in NYC [8, 9, 17, 41, 42, 49]. Interestingly, no studies were conducted in Europe.

While 25 models is not a large number compared to other fields such as cardiovascular disease, or COVID-19 [18, 55], there is a broad spectrum and heterogeneity of homelessness prediction models. Our review found an increase in the number of models being developed and in the size of the study population over time. Six studies targeted welfare applicants or recipients [8, 9, 17, 43, 46, 49], one study focused both on young adults emerging out from public assistance and unemployed workers [50]. Three studies created models in the context of US military personnel or veteran homelessness [16, 40, 44]. Two focused on emergency department patients [41, 42]. The other three didn’t focus on a specific population, one using utility data from service providers and two using data from health insurances [45, 47, 48].

As further evaluated in Appendix A, the most common types of predictors, using the original study classifications, were demographics, clinical history, homelessness history, human capital, housing conditions and criminal history. Only three studies combined geographical-level data with individual-level data [16, 44, 47], all others only included individual-level data. The use of geographical-level data alongside individual data is aligned with current research on the causality of homelessness, which shows a complex and multifaceted interaction between both individual and geographical factors [1–3, 6]. Geographical-level data describes structural factors such as overall poverty rate or income inequality, which differ between locations.

The methodological choices observed raise questions about potential biases. For example, reliance solely on individual-level data in most models may skew predictions toward personal risk factors while underemphasizing the broader structural influences captured by geographical-level data. Such an approach risks reinforcing a narrative that homelessness is predominantly an individual failing rather than a complex outcome of social and economic inequities. This is particularly concerning given the ethical implications associated with the potential misuse of these models, which could lead to discriminatory practices if predictive risk scores were used by stakeholders such as employers or credit agencies. While the ultimate aim of the studies is to prevent homelessness for individuals, it is important to consider that homelessness is associated with heavy stigma and discrimination, and homelessness research is often heavily influenced by current politics [56].

Our review reveals a notable predominance of regression-based methods, with 14 of 15 studies employing logistic or Cox regression techniques, while seven studies also incorporated ML algorithms such as random forest [16, 40, 47, 48, 50], CART [42], super learner [44] and XGboost [48]. This juxtaposition highlights a tension in the field: while the application of ML techniques has been increasing [57] and have the potential to capture non-linear relationships and interactions, their “black box” nature raises concerns about transparency, interpretability and feasibility—key features for clinical or social service applications. In fact, several studies [16, 47, 50] opted for regression models as the final model precisely because these are more intuitive and amenable to the development of scoring systems for practitioners, even when ML approaches offered competitive performance.

Although we did not critically appraise the quality of each included study, an important point is the limited use of calibration metrics; only two studies reported calibration measures [44, 47] despite calibration being essential for evaluating how well predicted probabilities reflect actual outcomes. Without adequate calibration and internal validation—employed in just nine of the studies [16, 17, 40, 42, 45, 47, 48, 50] —the real-world applicability of these models remains uncertain. Moreover, external validation helps with both quality assessment and proof of utility for other contexts [26, 58]. The scarcity of external validation (only two models were externally validated) limits our confidence in generalizing these models beyond the original study populations, which were often derived from highly specific settings (e.g., NYC homelessness prevention programs or veteran health administration data). We summarized the use of important quality markers in Appendix A.

Racial discrimination, studied as a proxy of ethnic/racial groups, has shown to be linked with homelessness [59]. Validation of the models in different ethnic/racial groups or on populations experiencing different levels of racial discrimination might be an important aspect for further research. Combining geographical data with individual data might help with accuracy and mitigate stigmatization through incorporating geographical data on marginalized and resource deprived communities, thus shifting the focus from the individuals to structural factors.

Ultimately, while both traditional regression and ML methods have demonstrated their utility in homelessness prediction, future work should focus on balancing performance with transparency and ensuring rigorous external validation. Further research might explore hybrid approaches that integrate ML’s capacity for handling complex data structures with the interpretability of regression models, alongside enhanced calibration techniques. Additionally, the field would benefit from standardizing reporting practices (e.g., through adherence to TRIPOD guidelines [60]) and incorporating ethical oversight, such as frameworks like embedded ethics in computer science and participatory research methods [61], to safeguard against potential misuse. For the development of new models and the evaluation of existing models, data privacy and consent of the future target populations should be thoroughly considered.

Concrete recommendations for the contexts in which the models should be evaluated are given in Appendix A.6.

US models for homelessness prevention services for welfare applicants [9, 17, 43, 49] have primarily been used as a triage tool within limited-capacity preventive programs. However, using risk models solely to decide service allocation raises ethical concerns [62, 63]. Rather than denying resources based on predicted risk, these models should serve as a complement to universal access: people actively seeking help should receive immediate support, while risk modeling can help identify at-risk individuals who might not otherwise apply for services.

An ideal implementation would integrate multiple models tailored to specific settings. For example, a municipality might utilize prediction models in emergency departments to screen youth transitioning out of public assistance or welfare recipients. In settings where comprehensive data are available, full-scale models could be applied—provided that robust ethical data protection measures are in place. In contrast, in contexts with limited data, simplified screening questionnaires could be used by social workers or clinicians to capture key risk indicators on site. People identified as “at-risk” through either method would then be referred to evidence-based, and well-funded prevention programs.

For instance, in a hypothetical scenario in Germany, a person applying for basic income support could consent to have their data assessed for homelessness risk. If the model flags high risk, the applicant would be offered additional support services, thereby ensuring that preventive resources reach not only those actively seeking help but also those silently at risk. The Homelessness Prevention Unit in Los Angeles, US, offers a compelling recent example using administrative data to identify people at risk of homelessness [64], of which only a very small number of the individuals identified as at high risk have applied for preventive services, underscoring the need for an integrative and outreaching approach.

Beyond guiding service allocation, risk prediction models may offer additional benefits. They can inform resource allocation by mapping risk at regional levels, enable integration into healthcare settings for early screening, and support ongoing evaluation of prevention programs. Such applications foster cross-sector collaboration, ultimately contributing to a more integrated, evidence-based approach to homelessness prevention.

### Limitations and strengths of this review

#### Strengths

We used a broad and previously validated filter search filter for prognostic prediction models [23]. Also, there was no time constraint for the publication date of the included studies. However, the homeless search string was more focused than the filter for prediction models and might have decreased sensitivity. Nonetheless, with the use of the citation chaser, only one extra study was identified, indicating a comprehensive result and good sensitivity.

#### Limitations

The search string was not translated into languages except German and English, and the data extraction was not fully done by two reviewers. The homelessness string was relatively focused and the search filter for prognostic models, which was developed to find studies which predicted health outcomes. The inclusion criteria were relatively narrow for a scoping review, because of the focus on models predicting future homelessness and on models that reported performance metrics. As this study was conducted as a scoping review, the included studies were not formally assessed for their methodological quality.

## Conclusion

In this scoping review, we provided an overview of prediction models for becoming homeless, their methodologies, settings and target populations, and the predictors they used. As only two of 25 models identified in this review were externally validated, we recommend that future research focuses on model evaluation. Also, future model developments should consider more thoroughly the ethical implications of their work as well as data protection. Prediction models for future homelessness have the potential to improve risk targeting and the effectiveness of preventive programs, but they can also be intentionally or unintentionally misused, and practical applications require careful consideration. Future research should carefully consider how to translate these models into real world contexts to help better identify and care for those at risk for homelessness.

## Supplementary Information

Additional File 1. Appendix A: supplemental results & discussion, search strategy, excluded references.

Additional File 2. Appendix B: extracted data.

Additional File 3. Appendix C: Prisma_Scr_checklist.

Additional File 4. Appendix D: R code, data analyses and visualization.

## Data Availability

All data generated or analyzed during this study is included in this published article and its supplementary information files.

## Abbreviations

AUC: Area under the receiver operator characteristic curve
CART: Classification and Regression Tree
CHARMS: CHecklist for critical Appraisal and data extraction for systematic Reviews of prediction Modelling Studies
ICD: International Classification of Diseases
HUD: US Department for Housing and Urban Development
NYC: New York City
PRISMA: Preferred Reporting Items for Systematic reviews and Meta-Analyses
PRISMA-ScR: Preferred Reporting Items for Systematic reviews and Meta-Analyses extension for Scoping Reviews
ROC: Receiver Operating Curve
TRIPOD: Transparent reporting of multivariable prediction models for individual prognosis or diagnosis
TRIPOD-SRMA: Transparent reporting of multivariable prediction models for individual prognosis or diagnosis: checklist for systematic reviews and meta-analyses
WHO: World Health Organization

## Glossary

Prediction model: A risk prediction model uses a selected number of variables to predict an outcome. For example, a prediction model may aim to predict the probability of a person having a stroke in the next ten years. The predictors in the model may or may not have a causal relationship with the outcome of interest. In the context of homelessness, the ‘number of times a service shut-off arrangement was established for a person’ [45] may be a good predictor of homelessness without a causal relationship. However, financial distress would be a causal predictor and the service shut-off a proxy. Prediction models are evaluated by their overall predictive performance [14, 65].
Model calibration: Measures how well the predicted and actual probabilities match [14].
Model discrimination: Measures how well the model distinguishes between those who develop the outcome and those who do not [14].
Model validation: Internal validation uses techniques to assess internal validity and make the prediction model usable outside of the development data and thereby reduces “overfitting.” Overfitted models describe the training data so exactly that they perform poorly on other data. External validation estimates the performance of the model in different individuals and assesses generalizability. External validation can be conducted at a later time or in a different population from individuals included in the development set [14].
