# Supplementary material for: Homelessness prediction models in high-income countries: a scoping review
Source: BMC Public Health. 2025 Nov 17;25:3964. doi: 10.1186/s12889-025-24855-x (PMC12621412; doi:10.1186/s12889-025-24855-x)
Supplement: Supplementary file 2 — Additional File 2. Appendix B: extracted data. [file 12889_2025_24855_MOESM2_ESM.pdf]

Appendix A

Table of Contents

A.1.Search strings....2

A.2. Inclusion/exclusion criteria....4

A.3. Data extraction form....5

A.4 Supplemental Table 1: Study definitions used....8

A. 5. Most common predictors....9

A.6. Quality measures....11

A.7. External Validation recommendations....12

A.8.1. Included studies....14

A.8.2. Fulltext excluded with reasons....15

A.1.Search strings

MEDLINE via Ovid (1946 to April 24, 2024)

1	Validat$.mp. or Predict$.ti. or Rule$.mp. or (Predict$ and (Outcome$ or Risk$ or Model$)).mp. or ((History or Variable$ or Criteria or Scor$ or Characteristic$ or Finding$ or Factor$) and (Predict$ or Model$ or Decision$ or Identif$ or Prognos$)).mp. or (Decision$.mp. and ((Model$ or Clinical$).mp. or Logistic Models/)) or (Prognostic and (History or Variable$ or Criteria or Scor$ or Characteristic$ or Finding$ or Factor$ or Model$)).mp.	6511727
2	stratification.mp. or exp "ROC curve"/ or discrimination.mp. or discriminate.mp. or c-statistic.mp. or "area under the curve".mp. or auc.mp. or calibration.mp. or indices.mp. or algorithm.mp. or multivariable.mp.	1284108
3	1 or 2	7163190
4	(homeless* or "ill-housed Persons/" or "homeless persons/" or unhous*)	17264
5	3 and 4	6061

Cochrane library (searched April 24, 2024)

((Validat?:ti,ab,kw OR Predict?:ti OR Rule?:ti,ab,kw OR (Predict?:ti,ab,kw AND (Outcome?:ti,ab,kw OR Risk?:ti,ab,kw OR Model?:ti,ab,kw)) OR ((History:ti,ab,kw OR Variable?:ti,ab,kw OR Criteria:ti,ab,kw OR Scor?:ti,ab,kw OR Characteristic?:ti,ab,kw OR Finding?:ti,ab,kw OR Factor?:ti,ab,kw) AND (Predict?:ti,ab,kw OR Model?:ti,ab,kw OR Decision?:ti,ab,kw OR Identif?:ti,ab,kw OR Prognos?:ti,ab,kw)) OR (Decision?:ti,ab,kw AND ((Model?:ti,ab,kw OR Clinical?:ti,ab,kw) OR [mh ^"Logistic Models"])) OR (Prognostic:ti,ab,kw AND (History:ti,ab,kw OR Variable?:ti,ab,kw OR Criteria:ti,ab,kw OR Scor?:ti,ab,kw OR Characteristic?:ti,ab,kw OR Finding?:ti,ab,kw OR Factor?:ti,ab,kw OR Model?:ti,ab,kw))) OR (stratification:ti,ab,kw OR [mh "ROC curve"] OR discrimination:ti,ab,kw OR discriminate:ti,ab,kw OR c-statistic:ti,ab,kw OR "area under the curve":ti,ab,kw OR auc:ti,ab,kw OR calibration:ti,ab,kw OR indices:ti,ab,kw OR algorithm:ti,ab,kw OR multivariable:ti,ab,kw)) AND ((homeless*) OR ("ill-housed Persons") OR ("homeless persons") OR (unhous*))

Web of science (searched April 24, 2024)

((Validat? OR Predict? OR Rule? OR (Predict? AND (Outcome? OR Risk? OR Model?)) OR ((History OR Variable? OR Criteria OR Scor? OR Characteristic? OR Finding? OR Factor?) AND (Predict? OR Model? OR Decision? OR Identif? OR Prognos?)) OR (Decision? AND ((Model? OR Clinical?) OR "Logistic Models")) OR (Prognostic AND (History OR Variable? OR Criteria OR Scor? OR Characteristic? OR Finding? OR Factor? OR Model?))) OR (stratification OR "ROC curve" OR discrimination OR discriminate OR c-statistic OR "area under the curve" OR auc OR calibration OR indices OR algorithm OR multivariable)) AND ((homeless*) OR ("ill-housed Persons") OR ("homeless persons") OR (unhous*))

Base (searched April 24, 2024)

https://www.base-search.net/

Since Base is a search engine, not a subject database, the complex phrase was not applicaple. The following terms were used:

Prediction model homeless

A.2. Inclusion/exclusion criteria

Inclusion criteria

To be included in the review, a study needed to report the following:

- Prognostic prediction model development (main objective)
- Model performance indicator, like discrimination or calibration measures
- Population the model was trained upon
- Setting in which the model should be applied
- Predictors included

A study needed to report at least one of the following:

- Model discrimination: Receiver Operating Characteristic (ROC) curve, Area Under the Curve (AUC), or C-statistic
- Model calibration measure: calibration plot, calibration slope, Hosmer-Lemeshow test
- Handling of missing data
- Internal Validation Method: bootstrapping, crossvalidation
- External Validation types and methods

Exclusion criteria

Studies were excluded if they focused on any of the following criteria:

- Diagnostic prediction model (detecting current homelessness)
- Prediction model for exiting homelessness
- Prediction model for leaving homelessness, predicting the probability of a homeless person to find adequate long-term housing
- Prediction of homeless prevalence
- Studies that investigate 'predictors' but the aim (at least underlying aim) is not developing/validating a prognostic risk model. Often these studies have a causal aim. Or the aim to identify the utility of singular predictor, which has already been reviewed (Nilsson et al., 2019)
- Studies with a non nested case control design, as they don’t allow for absolute risks to be calculated (Moons et al., 2014)

- Studies with a cross-sectional design, as these are used for diagnostic model development (Moons et al., 2014)

No time or language constraints were applied, articles in languages other than English and German were translated via DeepL, grey literature such as master theses or dissertations were also included.

A.3. Data extraction form

**Data Extraction Form**	
Author:	
Year:	
Data Source:	
Recruitment Method:	
Recruitment Dates:	
Study Setting:	
Study Region:	
Study Sites:	
Criteria for Inclusion:	
Criteria for Exclusion:	
Participant Age:	
Participant Gender:	
Study Dates:	
Outcome:	
Outcome Definition:	

Type of Outcome:	
Time to Outcome:	
Number of Candidate Predictors:	
type_of_c_predictors:	
Number of Participants:	
Number of Outcome Events:	
Number of Events per Variable:	
Number of Participants with Missing Value:	
Missing Data Handling:	
Modeling Method:	
Candidate Predictor Selection:	
Selection Predictors During Modeling:	
Shrinkage Predictor Weights:	
Calibration measure:	
discrimination_measure:	
classification_measure:	
other_performance_measures:	
Internal Validation Method:	
External Validation:	
Model Adjustment:	

Number of Final Predictors:	
final_predictors_type:	
final_predictors:	
Final Model Coefficients/Weights:	
Final Model Intercept Included:	
Alternative Model Presentation:	
Interpretation:	
Referencing O'Flaherty	
Referencing Shinn et al., (2013)	
Data Extraction Process:	
Additional Notes:	

A.4 Supplemental Table 1: Study definitions used

Table 1: Definitions used by the studies

Study	Outcome_definition	Type of Definition used
Brignone et al., 2018	"ICD-9-CM) code of V60.0 (indicating "lack of housing"	ICD 9
Byrne et al., 2022	shelter entry	NYC
Doran et al., 2021	shelter entry	NYC
Greer et al., 2014	1) literal homelessness, 2) literal homelessness or imminent housing loss (termed threatened homelessness), and 3) literal homelessness, imminent housing loss, or unstable housing (termed any housing instability	HUD+ETHOS
Greer et al., 2016	shelter entry	NYC
Koh et al., 2022	self reported	self report
Middleton et al.,2023	category1= literally homeless (HUD)	HUD
Mullen et al., 2022	shelter application	NYC
O'Flaherty et al., 2018	"Literal homelessness includes sleeping rough, squatting in abandoned buildings, or living in emergency or crisis accommodation."	HUD+Australia
Rodriguez et al., 2023	ehr meausures, discharge documentation, icd10 diagnostic, address, financial assistance	ICD 10+self report
Shahidi et al., 2023	na	na
Shinn et al., 1998	shelter entry	NYC
Shinn et al., 2013	shelter entry	NYC
Toros et al., 2019	Unemployed model=a person becoming homeless more than once or continuously for 12 or more months within three years after becoming unemployed,<br><br>young adults model=a person who became homeless more than once or continuously for 12 or more months within three years after becoming a young adult while receiving public assistance.	own
Tsai et al., 2024	not having stable housing that you either own, rent, or stay in as part of a household.	own

A. 5. Most common predictors

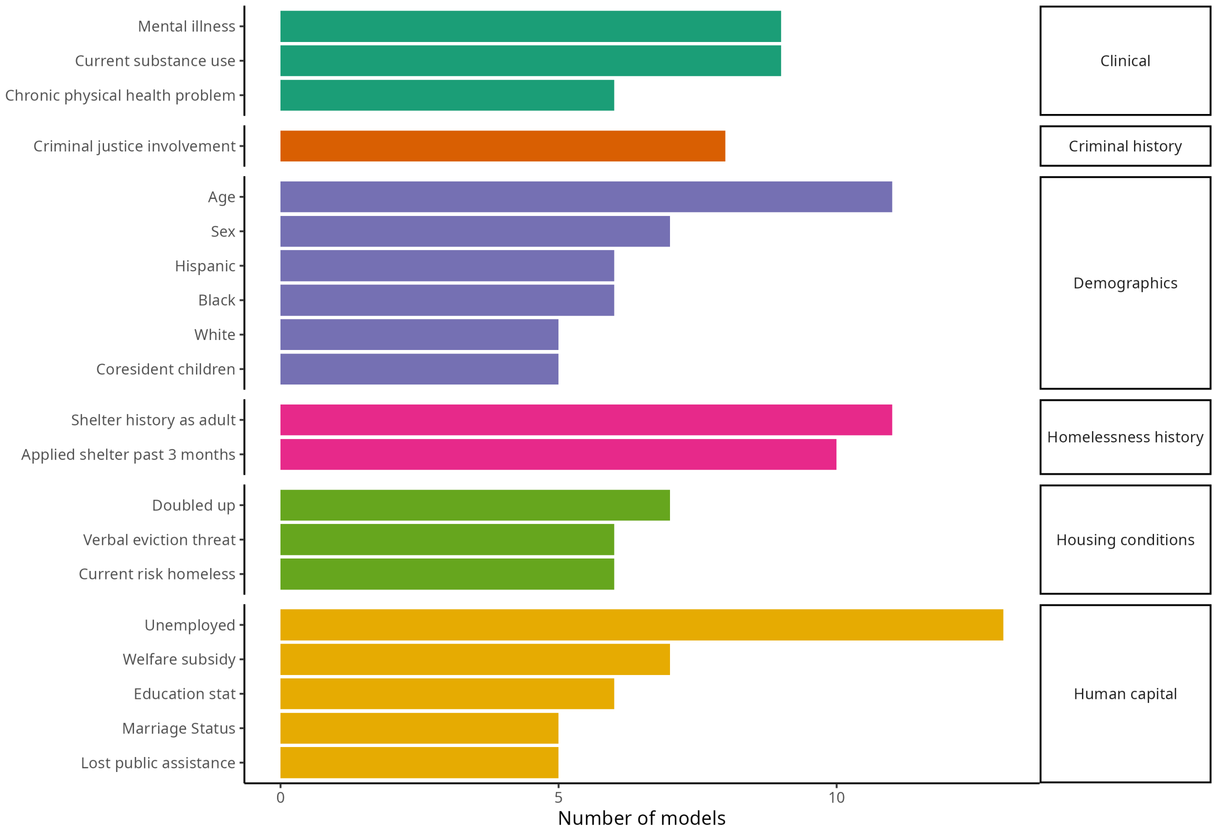

Supplemental figure 1: Most commonly used predictors

Supplemental **Figrue 1**shows the top 20 most common predictors used in the models of the included studies. The number of final predictors ranged from one to 47. The most common predictor was unemployment status, followed by age and shelter history as an adult. Other common predictors, also shown in **Error: Reference source not found**, were of the following types: demographics, clinical variables, housing conditions, human capital and criminal history. The types of predictors were taken from the studies without further interpretation. However, for the classification of the top 20 predictors, the most common classification for each predictor was used.

Two studies used predictors that most others did not: Byrne et al. [41] created a two-item screening tool that asked about the self-assessed risk of future homelessness, which only Doran et al. [42] and O'Flaherty et al. [44] used. Middleton et al. [2] used utility payment data, which none of the others used. A utility payment data predictor would be: 'Current amount owed to Avista and the city in all utility bills' or 'Number of times a service shutoff arrangement was established

for a person' [2]. All five models and all screening tools from Doran et al. [42] used past homelessness as a predictor. Tsai et al. [18], Koh et al. [19] and Rodriguez et al. [45] also used geographical-level predictors. Examples are 'county unemployment rate', 'percent widowed in county' [18]; 'county-level: very low motor vehicle rate', 'county level: high % of child food in-security' [19]; and 'eviction rate', 'county poverty rates' [45].

All extracted predictors can be found in **Appendix B.**

A.6. Quality measures

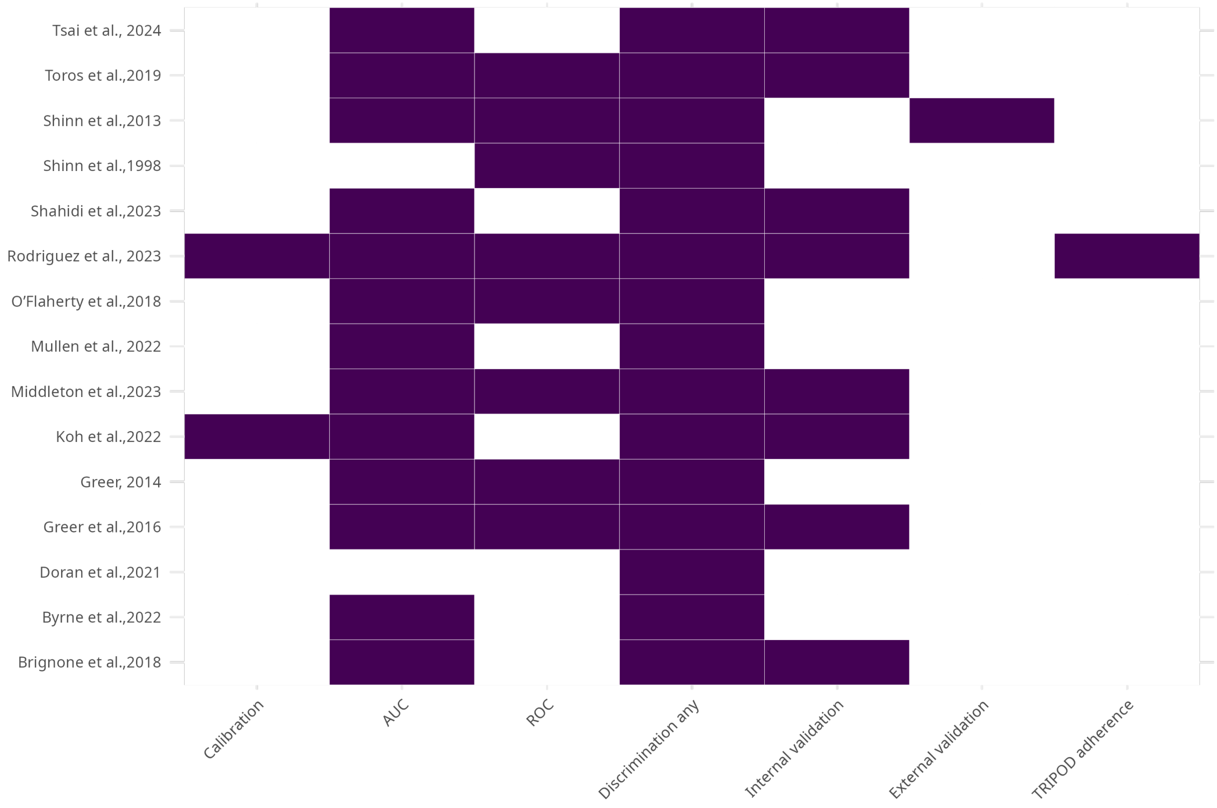

Supplemental Figure 2 shows the performance measures, implementation of validation, reporting according to the Transparent reporting of multivariable prediction models for individual prognosis or diagnosis (TRIPOD)-statement [54] and reporting of the final model coefficients. Two of the 15 studies reported calibration measures: calibration plot [43] and expected calibration error [19]. 14 studies reported area under the curve (AUC) as a discrimination statistic, Shinn et al. [45] used only Receiver Operating Curve (ROC) graphically as a discrimination statistic; while ROC was presented by eight studies [12, 17, 20, 40–43, 45] and by seven as a visual representation of the AUC [12, 17, 20, 40–43]. Internal validation was performed by nine studies [17–20, 37, 39, 41, 43, 44], with random data split being the most common method, which was used by five studies [17, 19, 20, 43, 44]. Only two models, the full model and the screening model developed by Shinn et al. [12] were externally validated, by Mullen et al. [13] in a different time setting. Also, only one study, Rodriguez et al. [43], reported their study ac-

cording to the TRIPOD statement, which would be applicable for all the described models [50]. The final model coefficients were reported by eleven out of 15 studies, Koh et al. [19], Doran et al. [39], Byrne et al. [38] and Brignone et al. [37] didn't report their model coefficients.

All studies reported that their models were performing well with an average AUC of 0,8 (SD=0,08) (own calculations). The full tables with all extracted model and study characteristics can be found in **Appendix B.**

A.7. External Validation recommendations

Since there are no models created in Europe, an appropriate next step could be to externally validate the US models within a European context. This is particularly relevant because Europe has made efforts to standardize homelessness definitions through ETHOS [10]. Adopting this standardized definition or establishing a validated classification that aligns corresponding categories across different definitions would be very valuable. It would enhance the reproducibility of scientific research and facilitate comparisons and applications not only of prediction models but potentially also of prevalence estimations and assessments of health statuses.

Following are the models and their contexts that should be externally validated to research their utility, as well as their predictive performance for other contexts. While some models might utilize very specific predictors and their utility outside their original context is debatable, they should at least be tested in a different time setting with new individuals in the same setting. Overfitting is an issue which is only partly resolved by internal validation and model performance measures can only be further evaluated with external validation [55].

In the context of homelessness prevention programs for welfare applicants in cities, the models developed by Shinn et al. [13] and Mullen et al. [14] for families and Greer et al. [18] for individuals have been used in practice [13, 14, 18].

Their usefulness for other countries and cities should be researched. Alternatively, they can be used as a basis for the development of new models or for updating the models, as well as researching a less questionable use case. It might be possible to use the models for people that receive general welfare benefits to identify those at risk for becoming homeless and refer them to a specified prevention program.

Tsai et al. [19] and Koh et al. [20] provide models for veterans that use both geographical-level and individual-level predictors and should be both externally validated in other countries or at differing time points in veterans. The study by Rodriguez et al. [46] also used both geographical-level and individual-level data, had by far the largest study population and is the only study

which was reported according to the TRIPOD [50] statement. Their model should be externally validated in other states or countries with extensive electronic health databases.

Both models from Toros et al. [21], which focused on young adults emerging out of public assistance and unemployed workers, should be externally validated first in other states of the US and then in other countries. Validation in other states of the US seems as a reasonable first step, since both models were developed in the context of the US welfare system.

From O'Flaherty et al.'s [45] study, the full model, using both public and private information should be validated. First in Australia, as the model is quite extensive and uses many predictors that are specific to the Australian welfare system and then in similar welfare systems.

Doran et al.'s [43] full models, context appropriate screening tools and the two-item screening model from Byrne et al. [42] should be externally validated in other emergency department settings. Especially the study from Byrne et al. [42] uses variables that are time and setting independent, assessing the self-assessed risk for homelessness and could be easily transferrable to other states and countries.

Middleton et al. [14] provide a unique model with promising utility, due to the availability of utility payment data in many municipalities. They combined anonymized utility payment data, which was provided by the city and the gas company, with records from emergency shelters. Since this data is available without the active participation of individuals at risk, it could be helpful to cover 'hard-to-reach' populations. Using this model could help to address the 'prevention paradox' [59], where those most at need are least likely to be reached by prevention efforts. However, from an ethical and data protection perspective, it is questionable how these models can be applied in practice. When and where would and could at-risk individuals be asked about their willingness to use their utility payment data? How could this data be protected from malevolent politicians or landlords?

The following models could be reviewed but had reporting or quality issues which would make external validation more difficult: Brignone et al. [41], Greer [44], [47].

Among the models reviewed, only those of Shinn et al. [13], Greer et al. [18] and Tsai et al. [19] reported their active use in practice. They state the adoption of their models by the NYC homelessness prevention programs and the Veteran Health administration, respectively, in their discussions. The NYC models have been shown to increase the efficiency of the prevention programs, in which they are utilized [13, 14, 18].

A.8.1. Included studies

Brignone, E., Fargo, J. D., Blais, R. K., & Gundlapalli, A. V. (2018). Applying Machine Learning to Linked Administrative and Clinical Data to Enhance the Detection of Homelessness among Vulnerable Veterans. AMIA ... Annual Symposium Proceedings. AMIA Symposium, 2018(101209213), 305–312.

Byrne, T., Hoang, M., Montgomery, A. E., Johns, E., Shinn, M., Mijanovich, T., Culhane, D., & Doran, K. M. (2022). Performance of 2 Single-Item Screening Questions to Identify Future Homelessness Among Emergency Department Patients. JAMA Network Open, 5(8), e2226691. https://doi.org/10.1001/jamanetworkopen.2022.26691

Doran, K. M., Johns, E., Zuiderveen, S., Shinn, M., Dinan, K., Schretzman, M., Gelberg, L., Culhane, D., Shelley, D., & Mijanovich, T. (2021). Development of a homelessness risk screening tool for emergency department patients. https://doi.org/10.1111/1475-6773.13886

Greer, A. L. (2014). Preventing homelessness in Alameda County, CA and New York City, NY: Investigating effectiveness and efficiency.

Greer, A. L., Shinn, M., Kwon, J., & Zuiderveen, S. (2016). Targeting Services to Individuals Most Likely to Enter Shelter: Evaluating the Efficiency of Homelessness Prevention. SOCIAL SERVICE REVIEW, 90(1), 130–155. https://doi.org/10.1086/686466

Koh, K. A., Montgomery, A. E., O'Brien, R. W., Kennedy, C. J., Luedtke, A., Sampson, N. A., Gildea, S. M., Hwang, I., King, A. J., Petriceks, A. H., Petukhova, M. V., Stein, M. B., Ursano, R. J., & Kessler, R. C. (2022). Predicting Homelessness Among U.S. Army Soldiers No Longer on Active Duty. https://doi.org/10.1016/j.amepre.2021.12.028

Middleton, C. D., Boynton, K., Lewis, D., & Oster, A. M. (2023). The value of utility payment history in predicting first-time homelessness. PloS One, 18(10), e0292305. https://doi.org/10.1371/journal.pone.0292305

Mullen, E. J., Ghesquiere, A., Dinan, K., Richard, M., Kealey, E., Zuiderveen, S., & Shinn, M. (2022). Periodic Evaluations of Risk Assessments: Identifying Families for Homelessness Prevention Services. Housing Policy Debate, 32(6), 915–939. https://doi.org/10.1080/10511482.2022.2077801

O'Flaherty, B., Scutella, R., & Tseng, Y.-P. (2018). Using Private Information to Predict Homelessness Entries: Evidence and Prospects. HOUSING POLICY DEBATE, 28(3), 368–392. https://doi.org/10.1080/10511482.2017.1367318

Rodriguez, L. A., Thomas, T. W., Finertie, H., Wiley, D., Dyer, W. T., Sanchez, P. E., Yassin, M., Banerjee, S., Adams, A., & Schmittdiel, J. A. (2023). Identifying Predictors of Homelessness Among Adults in a Large Integrated Health System in Northern California. https://doi.org/10.7812/TPP/22.096

Shahidi, F., MacDonald, M. E., Seitz, D., & Messier, G. (2023). The Effect of Epidemiological Cohort Creation on the Machine Learning Prediction of Homelessness and Police Interaction Outcomes Using Administrative Health Care Data. https://doi.org/10.48550/arXiv.2307.11211

Shinn, M., Greer, A. L., Bainbridge, J., Kwon, J., & Zuiderveen, S. (2013). Efficient Targeting of Homelessness Prevention Services for Families. American Journal of Public Health, 103(S2), S324–S330. https://doi.org/10.2105/AJPH.2013.301468

Shinn, M., Weitzman, B. C., Stojanovic, D., Knickman, J. R., Jiménez, L., Duchon, L., James, S., & Krantz, D. H. (1998). Predictors of homelessness among families in New York City: From shelter request to housing stability. American Journal of Public Health, 88(11), 1651–1657. https://doi.org/10.2105/AJPH.88.11.1651

Toros, H., Flaming, D., & Burns, P. (2019). Early Intervention to Prevent Persistent Homelessness: Predictive Models for Identifying Unemployed Workers and Young Adults who become Persistently Homeless. https://dx.doi.org/10.2139/ssrn.3370634

Tsai, J., Szymkowiak, D., Hooshyar, D., Gildea, S. M., Hwang, I., Kennedy, C. J., King, A. J., Koh, K. A., Luedtke, A., Marx, B. P., Montgomery, A. E., O'Brien, R. W., Petukhova, M. V., Sampson, N. A., Stein, M. B., Ursano, R. J., & Kessler, R. C. (2024). Predicting Homelessness Among Transitioning U.S. Army Soldiers. American Journal of Preventive Medicine, S0749379724000345. https://doi.org/10.1016/j.amepre.2024.01.018

A.8.2. Fulltext excluded with reasons

Access denied

Enich, M. (2023). *Identifying homelessness in Medicaid claims: A mixed-methods approach.* https://doi.org/10.7282/T3-498S-YH07

Gundlapalli, A. V., Redd, A., Carter, M. E., Palmer, M., Peterson, R., & Samore, M. H. (2014). Exploring patterns in resource utilization prior to the formal identification of homelessness in recently returned veterans. *Studies in Health Technology and Informatics*, *202*(ck1, 9214582), 265–268.

Catastrophic risk assessment

Mazumder, R. K., Enderami, S. A., Rosenheim, N., Sutley, E. J., Stanley, M., & Meyer, M. (2023). *Estimating long-term K-12 student homelessness after a catastrophic flood disaster.*

Merdjanoff, A. A., Abramson, D. M., Park, Y. S., & Piltch-Loeb, R. (2022). Disasters, Displacement, and Housing Instability: Estimating Time to Stable Housing 13 Years after Hurricane Katrina. *WEATHER CLIMATE AND SOCIETY*, *14*(2), 535–550. https://doi.org/10.1175/WCAS-D-21-0057.1

Ojo, W. E. (2019). *Measuring Affected Population Needs Using a Predictive Model for Health Resources Allocation During Disaster Response.*

Wilking, J., Roll, S., Kornbluh, M., & Donatello, R. (2023). Understanding Student Housing Insecurity and Homelessness: A Mixed Methods and Multi-variable Analysis. *JOURNAL OF STUDENT AFFAIRS RESEARCH AND PRACTICE*, *60*(5), 579–593. https://doi.org/10.1080/19496591.2022.2088292

Causal risk model

Bassuk, E. L., Buckner, J. C., Weinreb, L. F., Browne, A., Bassuk, S. S., Dawson, R., & Perloff, J. N. (1997). Homelessness in female-headed families: Childhood and adult risk and protective factors. *American Journal of Public Health*, *87*(2), 241–248. https://doi.org/10.2105/ajph.87.2.241

Boston, D. (2020). *Modeling the Relationship between the Housing First Approach and Homelessness.*

Brott, H., Kornbluh, M., Banfield, J., Boullion, A. M., & Incaudo, G. (2022). Leveraging research to inform prevention and intervention efforts: Identifying risk and protective

factors for rural and urban homeless families within transitional housing programs. *Journal of Community Psychology*, *50*(4), 1854–1874. https://doi.org/10.1002/jcop.22663
Brown, M., Chodzen, G., Mihelicova, M., & Collins, K. (2017). Applying a Time-Patterned Typology of Homelessness Among Individuals with Mental Illness. *American Journal of Community Psychology*, *59*(3–4), 306–315. https://doi.org/10.1002/ajcp.12140
Byrne, T., Munley, E. A., Fargo, J. D., Montgomery, A. E., & Culhane, D. P. (2013). NEW PERSPECTIVES ON COMMUNITY-LEVEL DETERMINANTS OF HOMELESSNESS. *JOURNAL OF URBAN AFFAIRS*, *35*(5), 607–625. https://doi.org/10.1111/j.1467-9906.2012.00643.x
Calvo, F., Fitzpatrick, S., Fabregas, C., Carbonell, X., & Turro-Garriga, O. (2020). Individuals experiencing chronic homelessness: A 10-year follow-up of a cohort in Spain. *Health & Social Care in the Community*, *28*(5), 1787–1794. https://doi.org/10.1111/hsc.13005
Caton, C. L. M., Dominguez, B., Schanzer, B., Hasin, D. S., Shrout, P. E., Felix, A., McQuistion, H., Opler, L. A., & Hsu, E. (2005). Risk factors for long-term homelessness: Findings from a longitudinal study of first-time homeless single adults. *American Journal of Public Health*, *95*(10), 1753–1759. https://doi.org/10.2105/AJPH.2005.063321
Chen, X., Cooper, I., & Rivier, J. (2022). Homelessness prevention and determinants of housing among first-time and recurrent emergency shelter users in Canada. *HOUSING STUDIES*, *37*(9), 1669–1685. https://doi.org/10.1080/02673037.2020.1865520
Cheng, T., Wood, E., Feng, C., Mathias, S., Montaner, J., Kerr, T., & DeBeck, K. (2013). Transitions into and out of homelessness among street-involved youth in a Canadian setting. *Health & Place*, *23*(din, 9510067), 122–127. https://doi.org/10.1016/j.healthplace.2013.06.003
Crawford, B. L., McDaniel, J., Moxley, D., Salehezadeh, Z., & Cahill, A. W. (2015). Factors Influencing Risk of Homelessness among Youth in Transition from Foster Care in Oklahoma: Implications for Reforming Independent Living Services and Opportunities. *Child Welfare*, *94*(1), 19–34.
Curtis, M. A., Corman, H., Noonan, K., & Reichman, N. E. (2014). Maternal depression as a risk factor for family homelessness. *American Journal of Public Health*, *104*(9), 1664–1670. https://doi.org/10.2105/AJPH.2014.301941
Czaderny, K. (2020). Risk factors for homelessness: A structural equation approach. *Journal of Community Psychology*, *48*(5), 1381–1394. https://doi.org/10.1002/jcop.22332
DiGuiseppi, G. T., Davis, J. P., Leightley, D., & Rice, E. (2020). Predictors of Adolescents' First Episode of Homelessness Following Substance Use Treatment. *The Journal of Adolescent Health : Official Publication of the Society for Adolescent Medicine*, *66*(4), 408–415. https://doi.org/10.1016/j.jadohealth.2019.11.312
Draheim, A. A., Kridel, M. M., Flinn, R. E., Ravoori, N., Brands, S., Mosley, C., Drescher, C. F., & Stepleman, L. M. (2023). Risk factors of homelessness among sexual and gender minorities in the Southeastern US. *JOURNAL OF SOCIAL DISTRESS AND THE HOMELESS*. https://doi.org/10.1080/10530789.2023.2276590
Dunga, S. H., & Grobler, W. C. J. (2018). *AN ANALYSIS OF THE SOCIO-ECONOMIC ANTECEDENTS OF HOUSING INSECURITY*.
Dworsky, A., Napolitano, L., & Courtney, M. (2013). Homelessness during the transition from foster care to adulthood. *American Journal of Public Health*, *103 Suppl 2*(1254074, 3xw), S318–23. https://doi.org/10.2105/AJPH.2013.301455
Dys, S., Steeves-Reece, A., & Carder, P. C. (2023). Lifelong Instability and Perceived Risk of Future Homelessness in Older Adults. *JOURNAL OF AGING AND ENVIRONMENT*, *37*(1), 46–64. https://doi.org/10.1080/26892618.2021.2001706
Embry, L. E., Vander Stoep, A. V., Evens, C., Ryan, K. D., & Pollock, A. (2000). Risk factors for homelessness in adolescents released from psychiatric residential treatment. *Journal*

of the American Academy of Child and Adolescent Psychiatry, *39*(10), 1293–1299. https://doi.org/10.1097/00004583-200010000-00017
Fargo, J., Metraux, S., Byrne, T., Munley, E., Montgomery, A. E., Jones, H., Sheldon, G., Kane, V., & Culhane, D. (2012). Prevalence and risk of homelessness among US veterans. *Preventing Chronic Disease*, *9*(101205018), E45-undefined.
Fothergill, K. E., Doherty, E. E., Robertson, J. A., & Ensminger, M. E. (2012). A prospective study of childhood and adolescent antecedents of homelessness among a community population of African Americans. *Journal of Urban Health : Bulletin of the New York Academy of Medicine*, *89*(3), 432–446. https://doi.org/10.1007/s11524-011-9641-y
Galano, M. M., Hunter, E. C., Howell, K. H., Miller, L. E., & Graham-Bermann, S. A. (2013). Predicting shelter residence in women experiencing recent intimate partner violence. *Violence against Women*, *19*(4), 518–535. https://doi.org/10.1177/1077801213487056
Giano, Z., Williams, A., Hankey, C., Merrill, R., Lisnic, R., & Herring, A. (2020). Forty Years of Research on Predictors of Homelessness. *Community Mental Health Journal*, *56*(4), 692–709. https://doi.org/10.1007/s10597-019-00530-5
Heerde, J. A., Bailey, J. A., Kelly, A. B., McMorris, B. J., Patton, G. C., & Toumbourou, J. W. (2021). Life-course predictors of homelessness from adolescence into adulthood: A population-based cohort study. *Journal of Adolescence*, *91*(hwt, 7808986), 15–24. https://doi.org/10.1016/j.adolescence.2021.06.007
Herbert, C. W., Morenoff, J. D., & Harding, D. J. (2015). Homelessness and Housing Insecurity Among Former Prisoners. *The Russell Sage Foundation Journal of the Social Sciences : RSF*, *1*(2), 44–79. https://doi.org/10.7758/rsf.2015.1.2.04
Kemp, P. A., Neale, J., & Robertson, M. (2006). Homelessness among problem drug users: Prevalence, risk factors and trigger events. *Health & Social Care in the Community*, *14*(4), 319–328. https://doi.org/10.1111/j.1365-2524.2006.00624.x
Koegel, P., Melamid, E., & Burnam, m A. (1995). Childhood risk factors for homelessness among homeless adults. *American Journal of Public Health*, *85*(12), 1642–1649. https://doi.org/10.2105/ajph.85.12.1642
Kube, A., Das, S., & Fowler, P. J. (2019). *Allocating Interventions Based on Predicted Outcomes: A Case Study on Homelessness Services*.
Lio, G., Ghazzai, M., Haesebaert, F., Dubreucq, J., Verdoux, H., Quiles, C., Jaafari, N., Chereau-Boudet, I., Legros-Lafarge, E., Guillard-Bouhet, N., Massoubre, C., Gouache, B., Plasse, J., Barbalat, G., Franck, N., & Demily, C. (2022). Actionable Predictive Factors of Homelessness in a Psychiatric Population: Results from the REHABase Cohort Using a Machine Learning Approach. *International Journal of Environmental Research and Public Health*, *19*(19). https://doi.org/10.3390/ijerph191912268
McBride, T., Calsyn, R., Morse, G., Klinkenberg, W., & Allen, G. (1998). Duration of homeless spells among severely mentally ill individuals: A survival analysis. *JOURNAL OF COMMUNITY PSYCHOLOGY*, *26*(5), 473–490. https://doi.org/10.1002/(SICI)1520-6629(199809)26:5<473::AID-JCOP6>3.0.CO;2-S
McQuistion, H. L., Gorroochurn, P., Hsu, E., & Caton, C. L. M. (2014). Risk factors associated with recurrent homelessness after a first homeless episode. *Community Mental Health Journal*, *50*(5), 505–513. https://doi.org/10.1007/s10597-013-9608-4
Metraux, S., Clegg, L. X., Daigh, J. D., Culhane, D. P., & Kane, V. (2013). Risk factors for becoming homeless among a cohort of veterans who served in the era of the Iraq and Afghanistan conflicts. *American Journal of Public Health*, *103 Suppl 2*(1254074, 3xw), S255-61. https://doi.org/10.2105/AJPH.2013.301432
Montgomery, A. E., Cusack, M., Szymkowiak, D., Fargo, J., & O'Toole, T. (2017). Factors contributing to eviction from permanent supportive housing: Lessons from HUD-VASH. *Evaluation and Program Planning*, *61*(7801727, eob), 55–63. https://doi.org/10.1016/j.evalprogplan.2016.11.014

Moxley, V. B. A., Hoj, T. H., & Novilla, M. L. B. (2020). Predicting homelessness among individuals diagnosed with substance use disorders using local treatment records. *Addictive Behaviors*, *102*(2gw, 7603486), 106160–undefined. https://doi.org/10.1016/j.addbeh.2019.106160
Mulcahy, E., Szymkowiak, D., & Montgomery, A. E. (2021). Psychosocial Risk Factors for Transitions Into Housing Instability Among Women Veterans. *Journal of the American Board of Family Medicine : JABFM*, *34*(2), 387–391. https://doi.org/10.3122/jabfm.2021.02.200333
Munley, E. (2012). *Community-level predictors of family homelessness in the United States.*
Naifeh, J. A., Capaldi, V. F., Chu, C., King, A. J., Koh, K. A., Marx, B. P., Montgomery, A. E., O'Brien, R. W., Sampson, N. A., Stanley, I. H., Tsai, J., Vogt, D., Ursano, R. J., Stein, M. B., & Kessler, R. C. (2022). Prospective Associations of Military Discharge Characterization with Post-active Duty Suicide Attempts and Homelessness: Results from the Study to Assess Risk and Resilience in Servicemembers-Longitudinal Study (STARRS-LS). *Military Medicine*, *2984771r, n1a*. https://doi.org/10.1093/milmed/usac232
Nesmith, A. (2006). Predictors of running away from family foster care. *Child Welfare*, *85*(3), 585–609.
Nilsson, S. F., Laursen, T. M., Hjorthoj, C., & Nordentoft, M. (2019). Risk of homelessness after discharge from psychiatric wards in Denmark: A nationwide register-based cohort study. *Acta Psychiatrica Scandinavica*, *140*(5), 477–489. https://doi.org/10.1111/acps.13082
North, C. S., Pollio, D. E., Smith, E. M., & Spitznagel, E. L. (1998). Correlates of early onset and chronicity of homelessness in a large urban homeless population. *The Journal of Nervous and Mental Disease*, *186*(7), 393–400. https://doi.org/10.1097/00005053-199807000-00002
Olfson, M., Mechanic, D., Hansell, S., Boyer, C. A., & Walkup, J. (1999). Prediction of homelessness within three months of discharge among inpatients with schizophrenia. *Psychiatric Services (Washington, D.C.)*, *50*(5), 667–673. https://doi.org/10.1176/ps.50.5.667
Orwin, R. G., Scott, C. K., & Arieira, C. (2005). *Transitions through homelessness and factors that predict them: Three-year treatment outcomes.*
Pedersen, E. R., DiGuiseppi, G., D'Amico, E. J., Rodriguez, A., Tran, D. D., Jose, R., & Tucker, J. S. (2024). Predictors of Housing Trajectories Among Young Adults Experiencing Homelessness in Los Angeles. *The Journal of Behavioral Health Services & Research*, *51*(1), 31–43. https://doi.org/10.1007/s11414-023-09863-2
Petry, L., Hill, C., Milburn, N., & Rice, E. (2022). Who Is Couch-Surfing and Who Is on the Streets? Disparities Among Racial and Sexual Minority Youth in Experiences of Homelessness. *The Journal of Adolescent Health : Official Publication of the Society for Adolescent Medicine*, *70*(5), 743–750. https://doi.org/10.1016/j.jadohealth.2021.10.039
Phinney, R., Danziger, S., Pollack, H. A., & Seefeldt, K. (2007). Housing instability among current and former welfare recipients. *American Journal of Public Health*, *97*(5), 832–837. https://doi.org/10.2105/AJPH.2005.082677
Pollio, D. E. (1997). The relationship between transience and current life situation in the homeless services-using population. *Social Work*, *42*(6), 541–551. https://doi.org/10.1093/sw/42.6.541
Rachlis, B. S., Wood, E., Zhang, R., Montaner, J. S. G., & Kerr, T. (2009). High rates of homelessness among a cohort of street-involved youth. *Health & Place*, *15*(1), 10–17. https://doi.org/10.1016/j.healthplace.2008.01.008
Remster, B. (2013). *Invisible men: A longitudinal analysis of homelessness among ex-inmates.*

Roebuck, M., Agha, A., Nelson, G., Distasio, J., Ecker, J., Hwang, S. W., Latimer, E., Purcell, S., Somers, J. M., Tsemberis, S., & Aubry, T. (2024). Predictors of housing instability and stability among housing first participants: A 24-month study. *JOURNAL OF SOCIAL DISTRESS AND THE HOMELESS*, *33*(1), 220–230. https://doi.org/10.1080/10530789.2023.2174565

Rosenthal, D., Rotheram-Borus, M. J., Batterham, P., Mallett, S., Rice, E., & Milburn, N. G. (2007). Housing stability over two years and HIV risk among newly homeless youth. *AIDS and Behavior*, *11*(6), 831–841. https://doi.org/10.1007/s10461-007-9235-6

Rye, S., & Aktas, E. (2023). *A Rule-Based Predictive Model for Estimating Human Impact Data in Natural Onset Disasters—The Case of a PRED Model.*

Shah, M. F., Liu, Q., Mark Eddy, J., Barkan, S., Marshall, D., Mancuso, D., Lucenko, B., & Huber, A. (2017). *Predicting Homelessness among Emerging Adults Aging Out of Foster Care.*

Shinn, M., Gottlieb, J., Wett, J. L., Bahl, A., Cohen, A., & Baron Ellis, D. (2007). Predictors of homelessness among older adults in New York city: Disability, economic, human and social capital and stressful events. *Journal of Health Psychology*, *12*(5), 696–708. https://doi.org/10.1177/1359105307080581

Slesnick, N., Guo, X., Brakenhoff, B., & Feng, X. (2013). *Two-year predictors of runaway and homeless episodes following shelter services among substance abusing adolescents.*

Sneddon, D. (2019). *Community-level and Individual-level Predictors of Variation in Rates of Homelessness among Youth Transitioning Out of Foster Care.*

Sosin, M. (2003). Explaining adult homelessness in the US by stratification or situation. *JOURNAL OF COMMUNITY & APPLIED SOCIAL PSYCHOLOGY*, *13*(2), 91–104. https://doi.org/10.1002/casp.716

Struble, C. A. (2021). *Understanding Developmental Trajectories of At-Risk Adolescents Transitioning into Emerging Adulthood.*

Sznajder-Murray, B., Jang, J. B., Slesnick, N., & Snyder, A. (2015). Longitudinal Predictors of Homelessness: Findings from the National Longitudinal Survey of Youth-97. *Journal of Youth Studies*, *18*(8), 1015–1034. https://doi.org/10.1080/13676261.2015.1020930

Treglia, D. A. (2016). *Using Positive Psychology to Explain Shelter Use: A Study of Homeless Families in New York City.*

Tsai, J., & Rosenheck, R. A. (2013). Conduct disorder behaviors, childhood family instability, and childhood abuse as predictors of severity of adult homelessness among American veterans. *Social Psychiatry and Psychiatric Epidemiology*, *48*(3), 477–486. https://doi.org/10.1007/s00127-012-0551-4

Tucker, J. S., Davis, J. P., Perez, L. G., Klein, D. J., & D'Amico, E. J. (2022). Late Adolescent Predictors of Homelessness and Food Insecurity During Emerging Adulthood. *The Journal of Adolescent Health : Official Publication of the Society for Adolescent Medicine*, *70*(5), 736–742. https://doi.org/10.1016/j.jadohealth.2021.10.035

van den Bree, M. B. M., Shelton, K., Bonner, A., Moss, S., Thomas, H., & Taylor, P. J. (2009). A longitudinal population-based study of factors in adolescence predicting homelessness in young adulthood. *The Journal of Adolescent Health : Official Publication of the Society for Adolescent Medicine*, *45*(6), 571–578. https://doi.org/10.1016/j.jadohealth.2009.03.027

van Laere, I. R., de Wit, M. A., & Klazinga, N. S. (2009). Pathways into homelessness: Recently homeless adults problems and service use before and after becoming homeless in Amsterdam. *BMC Public Health*, *9*(100968562), 3-undefined. https://doi.org/10.1186/1471-2458-9-3

Verissimo, A. D. O., Henley, N., Gee, G. C., Davis, C., & Grella, C. (2023). Homelessness and discrimination among US adults: The role of intersectionality. *JOURNAL OF SOCIAL*

DISTRESS AND THE HOMELESS, *32*(1), 1–15. https://doi.org/10.1080/10530789.2021.1935650
Washington, G. E. (2007). *What combination of factors is associated with length of homelessness?*
Weitzman, B. C., Knickman, J. R., & Shinn, M. (1992). Predictors of shelter use among low-income families: Psychiatric history, substance abuse, and victimization. *American Journal of Public Health*, *82*(11), 1547–1550. https://doi.org/10.2105/ajph.82.11.1547
Zhao, E. (2023). The key factors contributing to the persistence of homelessness. *INTERNATIONAL JOURNAL OF SUSTAINABLE DEVELOPMENT AND WORLD ECOLOGY*, *30*(1), 1–5. https://doi.org/10.1080/13504509.2022.2120109

Descriptive models

Adams, E. N., Clark, H. M., Galano, M. M., Stein, S. F., Grogan-Kaylor, A., & Graham-Bermann, S. (2021). Predictors of Housing Instability in Women Who Have Experienced Intimate Partner Violence. *Journal of Interpersonal Violence*, *36*(7–8), 3459–3481. https://doi.org/10.1177/0886260518777001
Aubry, T., Klodawsky, F., & Coulombe, D. (2012). Comparing the housing trajectories of different classes within a diverse homeless population. *American Journal of Community Psychology*, *49*(1–2), 142–155. https://doi.org/10.1007/s10464-011-9444-z
Barile, J. P., Pruitt, A. S., & Parker, J. L. (2018). A latent class analysis of self-identified reasons for experiencing homelessness: Opportunities for prevention. *JOURNAL OF COMMUNITY & APPLIED SOCIAL PSYCHOLOGY*, *28*(2), 94–107. https://doi.org/10.1002/casp.2343
Bearsley-Smith, C. A., Bond, L. M., Littlefield, L., & Thomas, L. R. (2008). The psychosocial profile of adolescent risk of homelessness. *European Child & Adolescent Psychiatry*, *17*(4), 226–234. https://doi.org/10.1007/s00787-007-0657-5
Belcher, J., & Toomey, B. G. (1988). Relationship between the deinstitutionalization model, psychiatric disability, and homelessness. *Health & Social Work*, *13*(2), 145–153. https://doi.org/10.1093/hsw/13.2.145
Blackford, K., Crawford, G., McCausland, K., & Zhao, Y. (2023). Describing homelessness risk among people from culturally and linguistically diverse backgrounds in Western Australia: A cluster analysis approach. *Health Promotion Journal of Australia : Official Journal of Australian Association of Health Promotion Professionals*, *34*(4), 953–962. https://doi.org/10.1002/hpja.704
Blue-Campbell, L. (2018). *Biopsychosocial Risk Associated with Homelessness amongst Baltimore City Veterans.*
Bramley, G., Fitzpatrick, S., & Sosenko, F. (2020). Mapping the “hard edges” of disadvantage in England: Adults involved in homelessness, substance misuse, and offending. *GEOGRAPHICAL JOURNAL*, *186*(4), 390–402. https://doi.org/10.1111/geoj.12358
Brown, M., Vaclavik, D., Watson, D. P., & Wilka, E. (2017). Predictors of homeless services re-entry within a sample of adults receiving Homelessness Prevention and Rapid Re-Housing Program (HPRP) assistance. *Psychological Services*, *14*(2), 129–140. https://doi.org/10.1037/ser0000112
Byrne, T., Fargo, J. D., Montgomery, A. E., Roberts, C. B., Culhane, D. P., & Kane, V. (2015). Screening for Homelessness in the Veterans Health Administration: Monitoring Housing Stability through Repeat Screening. *Public Health Reports (Washington, D.C. : 1974)*, *130*(6), 684–692. https://doi.org/10.1177/003335491513000618
Chikwava, F., Cordier, R., Ferrante, A., O’Donnell, M., & Pakpahan, E. (2024). Trajectories of homelessness and association with mental health and substance use disorders among

young people transitioning from out-of-home care in Australia. *Child Abuse & Neglect*, *149*(can, 7801702), 106643-undefined. https://doi.org/10.1016/j.chiabu.2024.106643

Chikwava, F., O'Donnell, M., Ferrante, A., Pakpahan, E., & Cordier, R. (2022). Patterns of homelessness and housing instability and the relationship with mental health disorders among young people transitioning from out-of-home care: Retrospective cohort study using linked administrative data. *PloS One*, *17*(9), e0274196-undefined. https://doi.org/10.1371/journal.pone.0274196

Crume, H. J., Nurius, P. S., & Fleming, C. M. (2019). Cumulative Adversity Profiles Among Youth Experiencing Housing and Parental Care Instability. *Children and Youth Services Review*, *100*(8110100), 129–135. https://doi.org/10.1016/j.childyouth.2019.02.042

Farmer, G. L., Heyman, J. C., Kelly, P. L., & Leaman, T. L. (2021). Prevalence of Risk and Protective Factors for Homelessness among Youth in Foster Care. *CHILD WELFARE*, *99*(1), 1–23.

Fowler, P. J., Marcal, K. E., Zhang, J., Day, O., & Landsverk, J. (2017). Homelessness and Aging Out of Foster Care: A National Comparison of Child Welfare-Involved Adolescents. *Children and Youth Services Review*, *77*(8110100), 27–33. https://doi.org/10.1016/j.childyouth.2017.03.017

Fowler, P. J., Marcal, K. E., Zhang, J., Day, O., & Landsverk, J. (2019). Defining Homelessness in the Transition to Adulthood for Policy and Prevention. *JOURNAL OF CHILD AND FAMILY STUDIES*, *28*(11), 3051–3061. https://doi.org/10.1007/s10826-019-01480-y

Fowler, P. J., Toro, P. A., & Miles, B. W. (2009). Pathways to and from homelessness and associated psychosocial outcomes among adolescents leaving the foster care system. *American Journal of Public Health*, *99*(8), 1453–1458. https://doi.org/10.2105/AJPH.2008.142547

Gleason, K., Barile, J. P., & Baker, C. K. (2017). Describing Trajectories of Homeless Service Use in Hawai'i Using Latent Class Growth Analysis. *American Journal of Community Psychology*, *59*(1–2), 158–171. https://doi.org/10.1002/ajcp.12128

Hatchimonji, D. R., Herbers, J. E., Flatley, C., Treglia, D., & Cutuli, J. J. (2024). Student Homelessness in High School: Prevalence, Individual Characteristics, and Profiles of Risk and Multidomain Functioning. *FAMILIES IN SOCIETY-THE JOURNAL OF CONTEMPORARY SOCIAL SERVICES*. https://doi.org/10.1177/10443894231215522

Haupert, T. (2023). Do housing and neighborhood characteristics impact an individual's risk of homelessness? Evidence from New York City. *HOUSING STUDIES*, *38*(9), 1740–1759. https://doi.org/10.1080/02673037.2021.1982874

Hershberger, A. R., Sanders, J., Chick, C., Jessup, M., Hanlin, H., & Cyders, M. A. (2018). Predicting running away in girls who are victims of commercial sexual exploitation. *Child Abuse & Neglect*, *79*(can, 7801702), 269–278. https://doi.org/10.1016/j.chiabu.2018.02.023

Holtyn, A., Carlson, E., Jarvis, B., Fingerhood, M., & Silverman, K. (2017). Rates of homelessness and factors that predict them among homeless, alcohol-dependent adults in a therapeutic workplace clinical trial. *Drug and Alcohol Dependence*, *171*, e90-undefined. https://doi.org/10.1016/j.drugalcdep.2016.08.254

Jeanis, M. N., Fox, B. H., & Muniz, C. N. (2019). Revitalizing Profiles of Runaways: A Latent Class Analysis of Delinquent Runaway Youth. *CHILD AND ADOLESCENT SOCIAL WORK JOURNAL*, *36*(2), 171–187. https://doi.org/10.1007/s10560-018-0561-5

Keenan, LynnD. (1996). *Identifying risk factors for homelessness among people living with HIV disease*.

Lee, J. S., Gimm, G., Mohindroo, M., & Lever, L. (2023). Assessing Homelessness and Incarceration Among Youth Aging Out of Foster Care, by Type of Disability. *CHILD AND*

ADOLESCENT SOCIAL WORK JOURNAL, *40*(6), 743–759. https://doi.org/10.1007/s10560-022-00817-9
Lim, G., Melendez-Torres, G. J., Amos, N., Anderson, J., Norman, T., Power, J., Jones, J., & Bourne, A. (2023). Demographic predictors of experiences of homelessness among lesbian, gay, bisexual, trans, gender-diverse and queer-identifying (LGBTIQ) young people in Australia. *JOURNAL OF YOUTH STUDIES*. https://doi.org/10.1080/13676261.2023.2261864
Matta, J., Singh, V., Auten, T., & Sanjel, P. (2023). Inferred networks, machine learning, and health data. *PloS One*, *18*(1), e0280910-undefined. https://doi.org/10.1371/journal.pone.0280910
Orsi-Hunt, R., Clemens, E. V., Thibodeau, H., & Belcher, C. (2023). Young Adults with Lived Foster Care Experience Who Later Experience Houselessness: An Exploratory Latent Class Analysis. *International Journal on Child Maltreatment : Research, Policy and Practice*, *101734300*, 1–25. https://doi.org/10.1007/s42448-023-00160-1
Pettey, W. B. P., Toth, D. J. A., Redd, A., Carter, M. E., Samore, M. H., & Gundlapalli, A. V. (2016). Using network projections to explore co-incidence and context in large clinical datasets: Application to homelessness among U.S. Veterans. *Journal of Biomedical Informatics*, *61*(100970413, d2m), 203–213. https://doi.org/10.1016/j.jbi.2016.03.023
Remster, B. (2021). Homelessness among Formerly Incarcerated Men: Patterns and Predictors. *ANNALS OF THE AMERICAN ACADEMY OF POLITICAL AND SOCIAL SCIENCE*, *693*(1), 141–157. https://doi.org/10.1177/0002716221993447
Tsai, J., & Byrne, T. H. (2024). Rates and Predictors of Returns to Homelessness Among Veterans, 2018-2022. *American Journal of Preventive Medicine*, *66*(4), 590–597. https://doi.org/10.1016/j.amepre.2023.11.013

Diagnostic model

Annaa, O. J. (2023). *Early Identification of Youth at Risk of Long Term Emergency Homeless Shelter Use: An Evaluation of Interpretable Machine Learning models*.
Arce, K. (2018). *Predicting Client Housing Outcomes from Georgia's Homeless Management Information System with Hierarchical Generalized Linear Modeling*.
Aubry, T. (Tim), Duhoux, A. (Arnaud), Klodawsky, F. (Fran), Ecker, J. (John), & Hay, E. (Elizabeth). (2016). *A Longitudinal Study of Predictors of Housing Stability, Housing Quality, and Mental Health Functioning Among Single Homeless Individuals Staying in Emergency Shelters*.
Booth, R. G., Richard, L., Forchuk, C., & Shariff, S. Z. (2022). Utility, Limitations and Opportunities for Using Linked Health Administrative Data to Study Homelessness in Ontario. *Healthcare Quarterly (Toronto, Ont.)*, *24*(4), 7–10. https://doi.org/10.12927/hcq.2022.26718
Byrne, T., Montgomery, A. E., & Fargo, J. D. (2019). *Predictive modeling of housing instability and homelessness in the Veterans Health Administration.*
Conway, M., Keyhani, S., Christensen, L., South, B. R., Vali, M., Walter, L. C., Mowery, D. L., Abdelrahman, S., & Chapman, W. W. (2019). Moonstone: A novel natural language processing system for inferring social risk from clinical narratives. *Journal of Biomedical Semantics*, *10*(1), 6-undefined. https://doi.org/10.1186/s13326-019-0198-0

Divita, G., Carter, M. E., Tran, L.-T., Redd, D., Zeng, Q. T., Duvall, S., Samore, M. H., & Gundlapalli, A. V. (2016). v3NLP Framework: Tools to Build Applications for Extracting Concepts from Clinical Text. *EGEMS (Washington, DC)*, *4*(3), 1228-undefined. https://doi.org/10.13063/2327-9214.1228

Elkins, M., Farrell, L., & Fry, J. M. (2023). Homelessness and housing insecurity among youth in Australia: Sequence analysis of housing careers. *HOUSING STUDIES*. https://doi.org/10.1080/02673037.2023.2203081

Erickson, J., Abbott, K., & Susienka, L. (2018). Automatic address validation and health record review to identify homeless Social Security disability applicants. *Journal of Biomedical Informatics*, *82*(100970413, d2m), 41–46. https://doi.org/10.1016/j.jbi.2018.04.012

Fargo, J. D., Montgomery, A. E., Byrne, T., Brignone, E., Cusack, M., & Gundlapalli, A. V. (2017). Needles in a Haystack: Screening and Healthcare System Evidence for Homelessness. *Studies in Health Technology and Informatics*, *235*(ck1, 9214582), 574–578.

Hatef, E., Rouhizadeh, M., Nau, C., Xie, F., Rouillard, C., Abu-Nasser, M., Padilla, A., Lyons, L. J., Kharrazi, H., Weiner, J. P., & Roblin, D. (2022). *Development and assessment of a natural language processing model to identify residential instability in electronic health records' unstructured data: A comparison of 3 integrated healthcare delivery systems.*

Hollister, B. M., Restrepo, N. A., Farber-Eger, E., Crawford, D. C., Aldrich, M. C., & Non, A. (2017). DEVELOPMENT AND PERFORMANCE OF TEXT-MINING ALGORITHMS TO EXTRACT SOCIOECONOMIC STATUS FROM DE-IDENTIFIED ELECTRONIC HEALTH RECORDS. *Pacific Symposium on Biocomputing. Pacific Symposium on Biocomputing*, *22*(cwq, 9711271), 230–241. https://doi.org/10.1142/9789813207813_0023

Montgomery, A. E., Fargo, J. D., Kane, V., & Culhane, D. P. (2014). Development and validation of an instrument to assess imminent risk of homelessness among veterans. *Public Health Reports (Washington, D.C. : 1974)*, *129*(5), 428–436. https://doi.org/10.1177/003335491412900506

Peterson, R., Gundlapalli, A. V., Metraux, S., Carter, M. E., Palmer, M., Redd, A., Samore, M. H., & Fargo, J. D. (2015). Identifying Homelessness among Veterans Using VA Administrative Data: Opportunities to Expand Detection Criteria. *PloS One*, *10*(7), e0132664-undefined. https://doi.org/10.1371/journal.pone.0132664

Pourat, N., Yue, D., Chen, X., Zhou, W., & O'Masta, B. (2023). *Easy to use and validated predictive models to identify beneficiaries experiencing homelessness in Medicaid administrative data.*

Richard, L., Hwang, S. W., Forchuk, C., Nisenbaum, R., Clemens, K., Wiens, K., Booth, R., Azimaee, M., & Shariff, S. Z. (2019). Validation study of health administrative data algorithms to identify individuals experiencing homelessness and estimate population prevalence of homelessness in Ontario, Canada. *BMJ Open*, *9*(10), e030221-undefined. https://doi.org/10.1136/bmjopen-2019-030221

Rios, S., Meyer, S. B., Hirdes, J., Elliott, S., & Perlman, C. M. (2021). The development and validation of a marginalization index for inpatient psychiatry. *The International Journal of Social Psychiatry*, *67*(4), 324–334. https://doi.org/10.1177/0020764020950785

Tsai, M., Weintraub, R., Gee, L., & Kushel, M. (2005). Identifying homelessness at an urban public hospital: A moving target?. *Journal of Health Care for the Poor and Underserved*, *16*(2), 297–307. https://doi.org/10.1353/hpu.2005.0042

Xie, F., Wang, S., Viveros, L., Rich, A., Nguyen, H. Q., Padilla, A., Lyons, L., & Nau, C. L. (2023). Using natural language processing to identify the status of homelessness and housing instability among serious illness patients from clinical notes in an integrated healthcare system. *JAMIA Open*, *6*(3), ooad082-undefined. https://doi.org/10.1093/jamiaopen/ooad082

Yao, Z., Tsai, J., Liu, W., Levy, D. A., Druhl, E., Reisman, J. I., & Yu, H. (2023). *Automated identification of eviction status from electronic health record notes*.

Zamora-Resendiz, R., Oslin, D. W., Hooshyar, D., & Crivelli, S. (2024). *Using electronic health record metadata to predict housing instability amongst veterans*. https://doi.org/10.1016/j.pmedr.2023.102505

Ethical implications

Chhabra, M., Sorrentino, A. E., Cusack, M., Dichter, M. E., Montgomery, A. E., & True, G. (2019). Screening for Housing Instability: Providers' Reflections on Addressing a Social Determinant of Health. *Journal of General Internal Medicine*, *34*(7), 1213–1219. https://doi.org/10.1007/s11606-019-04895-x

Eubanks, V. (2018). High-Tech Homelessness. *AMERICAN SCIENTIST*, *106*(4), 230–233. https://doi.org/10.1511/2018.106.4.230

Kuo, T.-S., Shen, H., Geum, J., Jones, N., Hong, J., Zhu, H., & Holstein, K. (2023). Understanding Frontline Workers' and Unhoused Individuals' Perspectives on AI Used in Homeless Services. *Arxiv*.

Moon, E. S.-Y., & Guha, S. (2024). *A Human-Centered Review of Algorithms in Homelessness Research*.

Tracey, P., & Garcia, P. (2024). After automation: Homelessness prioritization algorithms and the future of care labor. *BIG DATA & SOCIETY*, *11*(1). https://doi.org/10.1177/20539517241239043

No performance metric

Motupalli, V. (2015). *Predicting Risk for Incidences of Homelessness Among Veterans of Iraq and Afghanistan*.

Not high-income country

Chowdhury, M. H., & Shuvo, M. M. H. (2023). *A Smart Prediction Model for Identifying Homeless People Using Machine Learning and Immediate Assistance System for Homeless People Using Android Application*.

Ordóñez Erazo, H. A., Bucheli Guerrero, V. A., & Ordoñez Quintero, C. C. (2023). *Calle A model based on the gradient boosting regressor to predict trends in the ratio of residence in relation to the age of homeless people in Colombia ; Un modelo basado gradient boosting regressor para predecir tendencias de razón de residencia en relación a la edad de los habitantes de la calle en Colombia*.

Tsurugi, Y., Eam, K. K., Eang, M. T., Uehara, R., Nakamura, Y., Murakami, K., Sugiyama, T., Yamada, N., & Ishikawa, N. (2011). Evaluation of collaborative tuberculosis and human immunodeficiency virus activities in Phnom Penh, Cambodia. *The International Journal of Tuberculosis and Lung Disease : The Official Journal of the International Union against Tuberculosis and Lung Disease*, *15*(11), 1535–i. https://doi.org/10.5588/ijtld.10.0455

Outcome not homelessness

Aldridge, R. W., Shaji, K., Hayward, A. C., & Abubakar, I. (2015). Accuracy of Probabilistic Linkage Using the Enhanced Matching System for Public Health and Epidemiological Studies. *PloS One*, *10*(8), e0136179-undefined. https://doi.org/10.1371/journal.pone.0136179

Blumberg, M. (2023). *Differential Sensitivity of Intraindividual Variability Dispersion and Global Cognition in the Prediction of Functional Outcomes and Premature Mortality in Precariously Housed and Homeless Adults*.

Clemenzi-Allen, A. A., Hebert, J., Reid, M. A., Mains, T., Hammer, H., Gandhi, M., Pratt, L., & Wesson, P. (2024). Interruptions in HIV and Behavioral Health Care for Criminal-Legal Involved People Living with HIV Following Implementation of Decarceration and Shelter in Place in San Francisco, California. *AIDS and Behavior*, *28*(3), 1093–1103. https://doi.org/10.1007/s10461-023-04221-x

Cronley, C., Cimino, A. N., Hohn, K., Davis, J., & Madden, E. (2016). Entering Prostitution in Adolescence: History of Youth Homelessness Predicts Earlier Entry. *JOURNAL OF AGGRESSION MALTREATMENT & TRAUMA*, *25*(9), 893–908. https://doi.org/10.1080/10926771.2016.1223246

Handcock, M. (2014). *Scalable Model-Based Inference for Social Networks from Complex Sampling Designs*.

Helderop, E., Ferguson-Colvin, K., Grubesic, T. H., & Bender, K. (2018). *Predicting Movement of Homeless Young Adults: Artificial Neural Networks and Generalized Linear Models*.

Honey, A., Arblaster, K., Nguyen, J., & Heard, R. (2022). Predicting Housing Related Delayed Discharge from Mental Health Inpatient Units: A Case Control Study. *Administration and Policy in Mental Health*, *49*(6), 962–972. https://doi.org/10.1007/s10488-022-01209-y

Lemming, M. R., & Calsyn, R. J. (2004). *Utility of the Behavioral Model in Predicting Service Utilization by Individuals Suffering from Severe Mental Illness and Homelessness*.

Lewis, M. A., & Ferguson, K. M. (2014). Predicting Methamphetamine Use of Homeless Youths Attending High School: Comparison of Decision Rules and Logistic Regression Classification Algorithms. *JOURNAL OF THE SOCIETY FOR SOCIAL WORK AND RESEARCH*, *5*(2), 211–231. https://doi.org/10.1086/676830

Michel, M. (2013). *Swoistość adaptabilności i przewidywanie rezyliencji w “młodych bezdomnych” w lokalnym systemie profilaktyki i resocjalizacji w kontekście ekologicznego modelu Urie Bronfenbrennera ; Adaptability and resilience predict with “youth homeless” group in local system on prevention in context of Urie Bronfenbrenner developmental ecological model*.

Moore, G., Hepworth, G., Weiland, T., Manias, E., Gerdtz, M. F., Kelaher, M., & Dunt, D. (2012). *Prospective validation of a predictive model that identifies homeless people at risk of re-presentation to the emergency department*.

Salem, B. E., Nyamathi, A., Brecht, M.-L., Phillips, L. R., Mentes, J. C., Sarkisian, C., & Stein, J. A. (2014). *Constructing and identifying predictors of frailty among homeless adults —A latent variable structural equations model approach*.

Tabar, M. (2023). *Mitigating Social Challenges Among Vulnerable Communities With Machine Learning*.

Usacheva, M. (2022). *Empirical Inquiry into the Multidimensional Adversity: Implications for Families Affected by Housing Instability*.

van der Laan, J., van Straaten, B., Boersma, S. N., Rodenburg, G., van de Mheen, D., & Wolf, J. R. L. M. (2018). Predicting homeless people’s perceived health after entering the social relief system in The Netherlands. *International Journal of Public Health*, *63*(2), 203–211. https://doi.org/10.1007/s00038-017-1026-x

WALLACE, A. S. (2018). *FROM EMERGENCY TO COMMUNITY: IMPLEMENTING A SOCIAL NEEDS ASSESSMENT AND REFERRAL INFRASTRUCTURE USING HEALTH INFORMATION TECHNOLOGY.*

Policy review

Burt, M. R., Pearson, C., & Montgomery, A. E. (2007). Community-wide strategies for preventing homelessness: Recent evidence. *The Journal of Primary Prevention*, *28*(3–4), 213–228. https://doi.org/10.1007/s10935-007-0094-8

Crane, M., Warnes, A. M., & Fu, R. (2006). Developing homelessness prevention practice: Combining research evidence and professional knowledge. *Health & Social Care in the Community*, *14*(2), 156–166. https://doi.org/10.1111/j.1365-2524.2006.00607.x

Das, S. (2021). *Local Justice and the Algorithmic Allocation of Societal Resources.* https://doi.org/10.48550/ARXIV.2112.01236

Kube, A. R., Das, S., & Fowler, P. J. (2023). Community- and data-driven homelessness prevention and service delivery: Optimizing for equity. *Journal of the American Medical Informatics Association : JAMIA*, *30*(6), 1032–1041. https://doi.org/10.1093/jamia/ocad052

Prevalence prediction

Bates, A. J. (2020). *Predictors of urban homeless rates.*

Blid, M., Gerdner, A., & Bergmark, Å. (n.d.). *Prediction of Homelessness and Housing Provisions in Swedish Municipalities.*

Dawkins, C. J. J. (2023). Homelessness and housing supply. *JOURNAL OF URBAN AFFAIRS.* https://doi.org/10.1080/07352166.2023.2168553

De Freitas, H. (2015). *Homelessness Prevention with the Crisis Ministry of Mercer County: A Data Analysis Approach.*

Deal, C., & Gonzales, G. (2023). Homelessness Among Sexual Minority Youth. *Pediatrics*, *152*(6). https://doi.org/10.1542/peds.2023-062227

Doran, K. M., Johns, E., Schretzman, M., Zuiderveen, S., Shinn, M., Gulati, R., Wittman, I., Culhane, D., Shelley, D., & Mijanovich, T. (2020). Homeless Shelter Entry in the Year After an Emergency Department Visit: Results From a Linked Data Analysis. *Annals of Emergency Medicine*, *76*(4), 462–467. https://doi.org/10.1016/j.annemergmed.2020.03.006

Fiedler, R., Schumman, N., & Hyndman, J. (2006). Hidden homelessness: An indicator-based approach for examining the geographies of recent immigrants at-risk of homelessness in Greater Vancouver. *CITIES*, *23*(3), 205–216. https://doi.org/10.1016/j.cities.2006.03.004

Forty, L. (2008). *DEVELOPING A HOMELESSNESS PREDICTION MODEL …*

Foulkes, M., & Newbold, K. (2005). Geographic mobility and residential instability in impoverished rural Illinois places. *ENVIRONMENT AND PLANNING A*, *37*(5), 845–860. https://doi.org/10.1068/a34211

Gleason, K. D., Dube, M., Bernier, E., & Martin, J. (2022). Using geographic information systems to assess community-level vulnerability to housing insecurity in rural areas. *Journal of Community Psychology*, *50*(4), 1993–2012. https://doi.org/10.1002/jcop.22589

Jiang, M. (2017). *System Dynamics Modelling for hidden family homelessness—A case study in Fort Collins, Colorado.*

Johnson, S. M. (2010). *Homelessness: Selecting mitigation strategies to address the needs of the ‘at risk’ population.*

Tan, J. (2020). *Using machine learning to identify populations at high risk for eviction as an indicator of homelessness*.
Tsai, J., Hoff, R. A., & Harpaz-Rotem, I. (2017). One-year incidence and predictors of homelessness among 300,000 U.S. Veterans seen in specialty mental health care. *Psychological Services*, *14*(2), 203–207. https://doi.org/10.1037/ser0000083
Vandegrift, C. (2017). *Homelessness and the Low Income Housing Ratio: An Agent-Based Model Exploring Systematic Homelessness.*

Project proposal

Aldern, C. (2019). *Analyzing Systemic Racial Disparities With Statistical Learning Models and HMIS Data ...*
CASEY, R. (2012). *DEVELOPMENT AND INITIAL VALIDATION OF THE HOMELESSNESS SEVERITY INDEX.*
Coffee, N. T., Wilson, L., & Spoehr, J. D. (2004). *An approach to the development of a Predictive Model of Homelessness*.
Das, S. (2019). *EAGER: AI-DCL: Exploratory research on the use of AI at the intersection of homelessness and child maltreatment*.
Das, S. (2021). *RI: Small: Efficient and Just Allocation of Scarce Societal Resources, and Applications to Homelessness.*
DORAN, K. (2016). *Addressing Homelessness and Substance Use in Emergency Department Patients.*
flinker, adeen. (2016). *Bridging the Gap Between Big Data and Social Services ...*
Garfield, M. (2020). *SCC-PG: Improving Service Delivery for the Homeless with Analytics and Process Modeling—Community Engagement and Capacity Building*.
GUNDLAPALLI, A. (2013). *CURRENT EVIDENCE AND EARLY WARNING INDICATORS OF HOMELESSNESS RISK AMONG VETERANS.*
Lee, M. K. (2016). *Collaborative Research: SOCIUS: Socially Responsible Smart Cities*.
MONTGOMERY, A. E. (2015). *Identifying and Measuring Risk for Homelessness among Veterans.*
Morton, M. (2022). Research on Adolescent Predictors of Young Adult Homelessness and Food Insecurity Reveals Opportunities for Prevention. *The Journal of Adolescent Health : Official Publication of the Society for Adolescent Medicine*, *70*(5), 699–700. https://doi.org/10.1016/j.jadohealth.2022.02.006
Morton, M. H. (2020). The Complex Predictors of Youth Homelessness. *The Journal of Adolescent Health : Official Publication of the Society for Adolescent Medicine*, *66*(4), 381–382. https://doi.org/10.1016/j.jadohealth.2020.01.003
Noble, C. (n.d.). *Using a Predictive Risk Model to Identify Youth at Risk for Homelessness (Lessons from the Field).*
Wilson, L., & Spoehr, J. D. (2003). *Towards a predictive model of homelessness in South Australia: Report 1: National and international practice*.

Target poluation already homelessness

Alexander-Eitzman, B., North, C. S., & Pollio, D. E. (2018). Transitions between Housing States among Urban Homeless Adults: A Bayesian Markov Model. *Journal of Urban*

Health : Bulletin of the New York Academy of Medicine, *95*(3), 423–430. https://doi.org/10.1007/s11524-018-0236-8
Byrne, T., & Tsai, J. (2022). *Actuarial prediction versus clinical prediction of exits from a national supported housing program.*
Cusack, M., Montgomery, A. E., Blonigen, D., Gabrielian, S., & Marsh, L. (2016). Veteran Returns to Homelessness Following Exits From Permanent Supportive Housing: Health and Supportive Services Use Proximal to Exit. *FAMILIES IN SOCIETY-THE JOURNAL OF CONTEMPORARY SOCIAL SERVICES*, *97*(3), 221–229. https://doi.org/10.1606/1044-3894.2016.97.23
Ecker, J., & Aubry, T. (2016). Individual, Housing, and Neighborhood Predictors of Psychological Integration Among Vulnerably Housed and Homeless Individuals. *American Journal of Community Psychology*, *58*(1–2), 111–122. https://doi.org/10.1002/ajcp.12066
Edens, E. L., Mares, A. S., Tsai, J., & Rosenheck, R. A. (2011). Does active substance use at housing entry impair outcomes in supported housing for chronically homeless persons? *Psychiatric Services (Washington, D.C.)*, *62*(2), 171–178. https://doi.org/10.1176/ps.62.2.pss6202_0171
Fisher, A., Mago, V., & Latimer, E. (2020). Simulating the Evolution of Homeless Populations in Canada Using Modified Deep Q-Learning (MDQL) and Modified Neural Fitted Q-Iteration (MNFQ) Algorithms. *IEEE ACCESS*, *8*, 92954–92968. https://doi.org/10.1109/ACCESS.2020.2994519
Fisher, C. C., Ortiz, L., Alemi, Q., & Malika, N. M. (2020). *Congregations Serving Homeless Populations: Examining Predictive Factors and Policy Implications.*
Glendening, Z. S. (2017). *Risk Models for Returns to Housing Instability Among Families Experiencing Homelessness.*
Jackson, K. K. (1998). *There's no place like home: An examination of the relative importance of factors predicting the duration of homelessness and housing stability following homeless exit for families with children.*
John, C., & G. Messier, G. (2022). A RULE SEARCH FRAMEWORK FOR THE EARLY IDENTIFICATION OF CHRONIC EMERGENCY HOMELESS SHELTER CLIENTS. *Arxiv.*
Jones, A. L., Thomas, R., Hedayati, D. O., Saba, S. K., Conley, J., & Gordon, A. J. (2018). *Patient Predictors and Utilization of Health Services within a Medical Home for Homeless Persons.*
Kaltsidis, G., Grenier, G., Cao, Z., Bertrand, K., & Fleury, M.-J. (2022). *Predictors of change in housing status over 12 months among individuals using emergency shelters, temporary housing or permanent housing in Quebec, Canada.*
Khayyatkhoshnevis, P., Choudhury, S., Latimer, E., & Mago, V. (2020). Smart City Response to Homelessness. *IEEE ACCESS*, *8*, 11380–11392. https://doi.org/10.1109/ACCESS.2020.2965557
Kithulgoda, C. I., Vaithianathan, R., & Culhane, D. P. (2022). *Predictive Risk Modeling to Identify Homeless Clients at Risk for Prioritizing Services using Routinely Collected Data.*
Kontokosta, C., Hong, B., Malik, A., Bellach, I. M., Huang, X., Korsberg, K., Perl, D., & Somvanshi, A. (2017). *Predictors of Re-admission for Homeless Families in New York City: The Case of the Win Shelter Network.*
Kube, A. (2022). *Data-Driven Decision-Making: Using Counterfactual Predictions to Allocate Scarce Homeless Services Fairly and Efficiently.*
Lettner, B. H., Doan, R. J., & Miettinen, A. W. (2016). Housing outcomes and predictors of success: The role of hospitalization in street outreach. *Journal of Psychiatric and Mental Health Nursing*, *23*(2), 98–107. https://doi.org/10.1111/jpm.12287
Malik, A. (2021). *Predicting Chronic Homelessness at an Emergency Homeless Shelter in Calgary using Neural Network models and Time-Stamped data records …*

Messier, G. G., Tutty, L., & John, C. (2021). The Best Thresholds for Rapid Identification of Episodic and Chronic Homeless Shelter Use. *Arxiv*.

Messier, G., John, C., & Malik, A. (2022). Predicting Chronic Homelessness: The Importance of Comparing Algorithms using Client Histories. *JOURNAL OF TECHNOLOGY IN HUMAN SERVICES*, *40*(2), 122–133. https://doi.org/10.1080/15228835.2021.1972502

Nakada, M. R. (2020). *Methods of Imputation and Data Merging to Predict Supportive Housing Outcomes for Homeless Families in San Francisco*.

Polimis, K. (2017). *Developing Computational Approaches to Investigate Health Inequalities*.

Rahman, K. S., & Chelmis, C. (2023). *Modeling and predicting individual transitions within the homelessness system*.

Rothwell, D. W., Sussman, T., Grenier, A., Mott, S., & Bourgeois-Guerin, V. (2017). Patterns of Shelter Use Among Men New to Homelessness in Later Life: Duration of Stay and Psychosocial Factors Related to Departure. *Journal of Applied Gerontology : The Official Journal of the Southern Gerontological Society*, *36*(1), 71–93. https://doi.org/10.1177/0733464815624154

Ruiz, M. A., & Dorritie, M. T. (2020). *Clinical Utility of the Minnesota Multiphasic Personality Inventory-2-Restructured Form (MMPI-2-RF) in a Residential Treatment Program for Homeless Individuals …*

Salim, K. B. (2020). *Examining the Reliability and Validity of the Second Version of the Vulnerability Index-Service Prioritization Decision Tool (VI-SPDAT) for Single Adults*.

Scherling, A. (2018). *Predicting Exits from Permanent Supportive Housing in Los Angeles*.

Suchting, R., Businelle, M. S., Hwang, S. W., Padhye, N. S., Yang, Y., & Maria, D. M. S. (2020). *Predicting Daily Sheltering Arrangements among Youth Experiencing Homelessness Using Diary Measurements Collected by Ecological Momentary Assessment*.

Taib, M., & Messier, G. G. (2024). *Efficient Observation Time Window Segmentation for Administrative Data Machine Learning*.

VanBerlo, B., Ross, M. A. S., Rivard, J., & Booker, R. (2021). Interpretable machine learning approaches to prediction of chronic homelessness. *ENGINEERING APPLICATIONS OF ARTIFICIAL INTELLIGENCE*, *102*. https://doi.org/10.1016/j.engappai.2021.104243

Veldhuizen, S., Adair, C. E., Methot, C., Kopp, B. C., O'Campo, P., Bourque, J., Streiner, D. L., & Goering, P. N. (2015). Patterns and predictors of attrition in a trial of a housing intervention for homeless people with mental illness. *Social Psychiatry and Psychiatric Epidemiology*, *50*(2), 195–202. https://doi.org/10.1007/s00127-014-0909-x

Wong, Y., Culhane, D., & Kuhn, R. (1997). Predictors of exit and reentry among family shelter users in New York City. *SOCIAL SERVICE REVIEW*, *71*(3), 441–462. https://doi.org/10.1086/604265

Citation chaser

No performance metric

Wachter, T. V., Bertrand, M., Pollack, H., Rountree, J., & Blackwell, B. (n.d.). *Predicting and Preventing Homelessness in Los Angeles*.

Diagnostic model

Byrne, T. B., Travis P. ;. Land, Thomas; Bernson, Dana; Hood, Maria-Elena; Kennedy-Perez, Cheryl; Monterrey, Rodrigo; Smelson, David A. ;. Dones, Marc; Bharel, Monica. (2020).

A classification model of homelessness using integrated administrative data: Implications for targeting interventions to improve the housing status, health and well-being of a highly vulnerable population. *PloS One*, *15*(8). https://doi.org/10.1371/journal.pone.0237905

Causal risk model

Benjaminsen, L. (2015). Homelessness in a Scandinavian welfare state: The risk of shelter use in the Danish adult population: *Urban Studies*, *53*(10). https://doi.org/10.1177/0042098015587818

Byrne, T. T., Dan; Culhane, Dennis P; Kuhn, John; Kane, Vincent. (2015). Predictors of Homelessness Among Families and Single Adults After Exit From Homelessness Prevention and Rapid Re-Housing Programs: Evidence From the Department of Veterans Affairs Supportive Services for Veteran Families Program. *Housing Policy Debate*, *26*(1). https://doi.org/10.1080/10511482.2015.1060249

Descriptive risk model

Farrell, D. C. ; K., Alexis; Parulkar, Ashwin; Preda, Matthew; Toledo-Liz, Marisol; Fuller, Renee. (2022). Reassessing Measures of Risk for Homelessness Among Participants of a New York City Homelessness Prevention Program. *SSRN Electronic Journal*, *NA*(NA). https://doi.org/10.2139/ssrn.4184200
