# Supplementary material for: Homelessness prediction models in high-income countries: a scoping review
Source: BMC Public Health. 2025 Nov 17;25:3964. doi: 10.1186/s12889-025-24855-x (PMC12621412; doi:10.1186/s12889-025-24855-x)
Supplement: Supplementary file 4 — Additional File 4. Appendix D: R code, data analyses and visualization. [file 12889_2025_24855_MOESM4_ESM.html]

Appendix D2, Figure and Table creation


## Table of contents

- Libraries
- Table 1: Study information
- Table 2 Model characteristics
- Figure 2 Model characteristics
- Figure 3 Most common predictors
- Supplemental Figure 1: Quality measures

# Appendix D2, Figure and Table creation

## Libraries

Libraries needed

```
library(tidyverse)
library(knitr)
library(readxl)
library(kableExtra)
library(ggplot2)
library(ggrepel)
library(RColorBrewer)
library(patchwork)
library(reshape2)
library(ggh4x)
library(ggbreak)
```

Import data.

```
study_data2 <- read_excel("/home/kun/Documents/R/Masterarbeit/Appendix/Appendix_B.xlsx", sheet=2, n_max=16)
model_data <- read_excel("/home/kun/Documents/R/Masterarbeit/Appendix/Appendix_B.xlsx", sheet=3)
```

# Table 1: Study information

Select relevant columns, make table with kable package

```
study_data_short <- study_data2 |>
  select(Study_id, Study_setting, Study_region, n_models, population, modeling_method)

kable(study_data_short) |>
  kable_styling(font_size = 8)
```

| Study\_id | Study\_setting | Study\_region | n\_models | population | modeling\_method |
| --- | --- | --- | --- | --- | --- |
| Tsai\_2024 | veteran health administration | Usa | 2 | us veterans | random forest, lasso regression |
| Middleton\_2023 | Spokane city | Washington | 2 | general population | binary logistic regression |
| Toros\_2019 | Los Angeles | california | 2 | young adults, unemployed workers | logistic regression, random forest |
| Shinn\_2013 | omelessness prevention program | new york city | 2 | welfare applicants (families) | cox regression |
| Shahidi\_2023 | Electronic health records | Calgary, Alberta | 1 | general population | linear regression, randomf forest, XGBoost |
| O’Flaherty\_2018 | Australian welfare program | Australia | 3 | welfare recipients | logistic regression? |
| Shinn\_1998 | homelessness prevention program | new york city | 1 | welfare applicants (families) | logistic regression |
| Greer\_2016 | homelessness prevention program | new york city | 2 | welfare applicants (individuals) | cox proportional hazards regression |
| Brignone\_2018 | veteran health administration | usa | 1 | us veterans | random forest |
| Doran\_2021 | emergency departement | new york city | 5 | patients | logistic, CART, LASSO |
| Byrne\_2022 | emergency departement | new york citiy | 1 | patients | logistic regression |
| Koh\_2022 | Veteran health administration | usa | 1 | us veterans | “0-fold cross-validation super learner |
| Mullen\_2022 | homelessness prevention program | new york city | 2 | welfare applicants (families) | cox regression |
| Greer\_2014 | homelessness prevention program | alameda county, california | 1 | welfare applicants (individuals) | cox regression |
| Rodriguez\_2023 | Electronic health records | northern california | 1 | general population | logisitic regression ,random forest |

# Table 2 Model characteristics

Repeat process for model characteristics

```
model_data_short <- model_data |>
  select(Study_id, model_name, Model_description, `Participant_gender_female_%`, time_to_outcome_, n_participants, n_final_predictors,type_final_predictor)
colnames(model_data_short) <- c(
  "Study ID", 
  "Model Name", 
  "Model description", 
  "Female %", 
  "Time to Outcome", 
  "No. Participants", 
  "No. of Predictors", 
  "Type of Final Predictor"
)
kable(model_data_short) |>
  kable_styling(font_size=7)
```

| Study ID | Model Name | Model description | Female % | Time to Outcome | No. Participants | No. of Predictors | Type of Final Predictor |
| --- | --- | --- | --- | --- | --- | --- | --- |
| Tsai\_2024 | Tsai\_stage1 | Identifies transitioning veterans at high risk of homelessness within 12 months of leaving the military. Uses indiviudal and geopgraphical data | 16 | 12 | 4790 | 10 | unemployment rate, widow rate, age rate, unemployed veterans rate, discharge status, |
| Tsai\_2024 | Tsai\_stage2 | To be used on individuals identified by the first model, is based on existing administrative data and a 10 question self assessment | 16 | 12 | 4790 | 10 | family status, religious, physical health, mental health, suicidal affiliation |
| Middleton\_2023 | Middleton\_general | Prediction of future homelessness based on utility payment data. General model | na | 12 | 86317 | 5 | number of persons at household, money owed, pay plan deviations |
| Middleton\_2023 | Middleton\_spokane | Model for spokane city, uses more specific data | na | 12 | 86318 | 14 | specific utilities not payed |
| Toros\_2019 | Toros\_young\_adults | Prediction of future homelessness for youth transitioning out of social services | na | 36 | 479111 | 22 | demographics, employment, homelessness history, health and behavioral health ,criminal justice, social services, foster care. |
| Toros\_2019 | Toros\_unemployed | Prediction of future homelessness for unemployed workers | na | 36 | 494584 | 43 | demographics, employment, homelessness history, health and behavioral health ,criminal justice, social services |
| Shinn\_2013 | Shinn13\_full\_model | Prediction of future shelter entry for families applying for homelessness prevention services. Shows that prediction models can make such programs more efficient. | 91.7 | 36 | 11105 | 41 | demographics, human capital, housing conditions, disability and criminal justice, interpersonal discord, childhood experiences, shelter history self reported, shelter history administrative data |
| Shinn\_2013 | Shinn13\_screening\_model | Questionnaire version of the model | 92.7 | 36 | 11106 | 15 | demographics, human capital, housing conditions, disability and criminal justice, interpersonal discord, childhood experiences, shelter history self reported, shelter history administrative data |
| Shahidi\_2023 | Shahidi | Comparison of predictive performance of homelessness prediction models using either a fixed cohort or a flexible cohort. | 36.2 | 24 | 237602 | 28 | Gender, age, emergency department information, clinic history visits, comorbidities |
| O’Flaherty\_2018 | O’Flaherty public\_private | Using only Public information | na | 3-12 | 1919 | 39 | housing conditions, homelessness history, human capital, clinical, interpersonal history, demographics, childhood experiences, criminal history |
| O’Flaherty\_2018 | O’Flaherty private | Using only Private information | na | 3-12 | 1919 | 39 | housing conditions, homelessness history, human capital, clinical, interpersonal history, criminal history |
| O’Flaherty\_2018 | O’Flaherty public | The use of private, not publicly available information, adds predictive values to homelessness prediction models | na | 3-12 | 1919 | 39 | demographics, human capital, childhood experiences, clinical, housing conditions |
| Shinn\_1998 | Shinn98\_full\_model | Old prediction model, original author interpretation is mainly causal | 95 | 60 | 563 | 21 | demographics, persistent poverty, disorder, social ties, housing |
| Greer\_2016 | Greer16\_full\_model | Prediction of future homelessness for individuals in New Yourk City | 38,1, nos, 45,5 s | 12-96 | 10220 | 37 | demographics, human capital, housing conditions, disability, interpersonal discord, childhood experiences, and shelter history. |
| Greer\_2016 | Greer16\_screening\_model | Questionnaire version of the model | 38,1, nos, 45,5 s | 12-96 | 10220 | 7 | demographics, human capital, housing conditions, disability, shelter history. |
| Brignone\_2018 | Brignone | Prediction of future homelessness for veterans | 7.8 | 1,3,12,12 | 25510 | na | demographic, military service, health status, and healthcare utilization characteristics. |
| Doran\_2021 | Doran logistic | Logistic regression model | 47.5 | 6 | 1993 | 12 | human capital, homelessness history, self assessed future risk, clinical, housing conditions, demographics |
| Doran\_2021 | D CART | CART model | 47.5 | 6 | 1993 | 9 | self assessed risk, human capital, housing conditions, clinical, criminal history, homelessness history, self assessed risk ,housing conditions clinical |
| Doran\_2021 | D CART+K | Cart model with h-fold cross validation | 47.5 | 6 | 1993 | 3 | human capital, homelessness history,demographics |
| Doran\_2021 | D BIS | Logistic model with Bayesion Information Criterion | 47.5 | 6 | 1993 | 1 | homelessness history |
| Doran\_2021 | D LASSO | Full 10-fold cross-validation with selection of variables using LASSO | 47.5 | 6 | 1993 | 6 | criminal history, homelessness history, self assessed risk, homelessness history |
| Byrne\_2022 | Byrne\_2\_item\_screening\_tool | Two item screening tool for emergency departement patients | 49 | 2, 6, 12 | 1919 | 2 | self assessed risk |
| Koh\_2022 | Koh | Prediction of future homelessness in US veterans | na | 12 | 16589 | 26 | mental health, adverse childhood experiences, lifetime traumas, geospatial variables |
| Mullen\_2022 | Mullen full model | External validation of Shinn\_2013 model | 90.5 | 36 | 48450 | 47 | sociodemographics, child-related, return to residence, housing items, childhood adversitiy |
| Mullen\_2022 | Mullen screening model | External validation of Shinn\_2013 model | 90.5 | 36 | 48450 | 47 | sociodemographics, child-related, return to residence, housing items, childhood adversitiy |
| Greer\_2014 | Greer14\_full\_model | Prediction of future homelessness for individuals in Alameda | na | Unclear, 36? | 2761 | 9 | demographics, human capital, housing conditions, shelter history |
| Rodriguez\_2023 | Rodriguez | Prediction model using electronic health records. To be used in inpatient, outpatient, or emergency department settings. | 52.8 | 12-24 | 2543503 | 26 | demographics, clinical, geographical-level |

# Figure 2 Model characteristics

```
#combine both tables 
combined <- left_join(study_data2, model_data, by="Study_id")

#create better names
combined$Study_region <- str_replace_all(combined$Study_region, "new york citiy", "new york city")
combined$Study_region<- str_replace_all(combined$Study_region, "Usa", "usa")
combined$Study_region<- str_replace_all(combined$Study_region, "northern california", "california")
combined$Study_region
```

```
 [1] "usa"                        "usa"                       
 [3] "Washington"                 "Washington"                
 [5] "california"                 "california"                
 [7] "new york city"              "new york city"             
 [9] "Calgary, Alberta"           "Australia"                 
[11] "Australia"                  "Australia"                 
[13] "new york city"              "new york city"             
[15] "new york city"              "usa"                       
[17] "new york city"              "new york city"             
[19] "new york city"              "new york city"             
[21] "new york city"              "new york city"             
[23] "usa"                        "new york city"             
[25] "new york city"              "alameda county, california"
[27] "california"
```

```
combined$Study_region <- str_replace_all(combined$Study_region, "usa", "US General" )
combined$Study_region <- str_replace_all(combined$Study_region, c("california"="US California", "alameda county, US California"= "US California", "Washington"="US Washington",  "Calgary, Alberta"= "Canada, Calgary", "new york city"= "US New York City") )
combined$population_m <- str_replace_all(combined$population_m, c("us veterans"="US military personnel"))

combined$model_name <- str_replace_all(combined$model_name, "Doran_(\\d+)_item_screening_tool", "Doran_\\1_item")
combined$model_name <- str_replace(combined$model_name, "Byrne_2_item_screening_tool", "Byrne_2_item")

#make sure all columns have the correct type

combined$n_participants <- as.numeric(combined$n_participants)
combined$population_m <- as.factor(combined$population_m)
combined$Study_region <- as.factor(combined$Study_region)
combined$Year <- as.numeric(combined$Year)
  

#make another table with relevant columns
plot_table <- combined |>
  select(Study_id,model_name, Year, Study_region, population_m, n_participants)

plot_table$Study_region
```

```
 [1] US General       US General       US Washington    US Washington   
 [5] US California    US California    US New York City US New York City
 [9] Canada, Calgary  Australia        Australia        Australia       
[13] US New York City US New York City US New York City US General      
[17] US New York City US New York City US New York City US New York City
[21] US New York City US New York City US General       US New York City
[25] US New York City US California    US California   
6 Levels: Australia Canada, Calgary US California ... US Washington
```

```
#define colorblind friendly color palette
brewer_palette <- brewer.pal(n = 8, name = "Dark2")


custom_colors <- c(
  "general population" = brewer_palette[1],           # Blue from the palette
  "patients" = brewer_palette[2],                     # Green from the palette
  "unemployed workers" = brewer_palette[8],           # Red from the palette
  "US military personnel" = brewer_palette[4],                  # Purple from the palette
  "welfare applicants (families)" = brewer_palette[5],# Orange from the palette
  "welfare applicants (individuals)" = brewer_palette[5], # Similar color for consistency
  "welfare recipients" = brewer_palette[5],           # Similar color for consistency
  "young adults" = brewer_palette[3]                  # Pink from the palette
)


p1 <- ggplot(plot_table, aes(x = Year, y = factor(Study_region, levels = c("US Washington", "US New York City", "US General", "US California", "Australia", "Canada, Calgary")), color = population_m, size = n_participants)) +
  ylab("Study_region") +
  geom_point(alpha = 0.5, position = position_jitter(width = 0.2, height = 0.2)) +
  geom_text_repel(aes(label = model_name, color = population_m), size = 3, max.overlaps = 15) +
  theme_classic() +
  scale_size_continuous(range = c(5, 50), breaks = c(50, 500, 5000, 50000)) +
  guides(size = guide_legend(title = "Number of Participants", keywidth = 0.5, keyheight = 0.5, label.theme = element_text(size = 8))) +
  labs(
    color = "Population",
    size = "Number of Participants",
    x = "Year",
    y = "Study Region"
  ) +
  scale_color_manual(values = custom_colors) 
p1
```

```
#works
p1+ scale_x_cut(c(1998), scales=20)
```

```
#is ok
p1+ scale_x_break(c(1998.5, 2013), scales = 10,  ticklabels = c(1998, 2013, 2014, 2016, 2018, 2019, 2021, 2022, 2023, 2024), space=1)
```

# Figure 3 Most common predictors

import data, type of predictor and predictor. Use sheet 9, because R likes tables oriented in the other direction. Corresponds to sheet B.4.1.extracted predictors coded.

```
type_final_p <- read_excel("/home/kun/Documents/R/Masterarbeit/Appendix/Appendix_B.xlsx", sheet=10)
final_p <- read_excel("/home/kun/Documents/R/Masterarbeit/Appendix/Appendix_B.xlsx", sheet=11)
```

```
#convert to 0 and 1, or present absent
final_p_01 <- final_p %>%
  mutate_at(vars(-model_name), ~ if_else(is.na(.), 0, 1))
#calculate sum, how often it was present
sum_row <- final_p_01|>
    summarise(across(2:151, sum))

#rename the columns to their correspoding entries (already done in sheet B.4.2.)

sum_row <- sum_row |>
  rename(Age= Demographic_1, Sex=Demographic_2, Indigenous=Demographic_3, Asian= Demographic_4 , Black=Demographic_5, Hispanic=Demographic_6, Missing_race= Demographic_7, Multiracial= Demographic_8, White= Demographic_9, American_Indian_Asian_Black_Multiple_vs_Other_Race=Demographic_10, Marriage_Status=Demographic_11, Coresident_children=Demographic_12, Pregnant=Demographic_18, Child_under_2=Demographic_20, Child_in_foster_care= Demographic_21, Protective_service_case_child=Demographic_22,education_stat=Human_capital_1, Unemployed=Human_capital_2, Welfare_subsidy= Human_capital_3, Lost_public_assistance=Human_capital_4, time_since_last_employment=Human_capital_5, Average_income_last_year=Human_capital_6, Maximum_income_last_year=Human_capital_7, Veteran_status=Human_capital_8, Junior_army_enlistment=Human_capital_9, Social_ties=Human_capital_10,Reintegration_into_community= Human_capital_11, Dept=Human_capital_13, current_risk_homeless=homelessness_history_1 , Shelter_history_as_adult = homelessness_history_2, returned_to_residence_after_institution_or_shelter_6months=homelessness_history_3, Applied_shelter_past_3months=homelessness_history_4, name_on_lease=housing_conditions_1, overcrowded=housing_conditions_2, doubled_up=housing_conditions_3, Verbal_eviction_threat=housing_conditions_4, Rent_over_50percent_income=housing_conditions_5, rental_arrea=housing_conditions_6, unsafe_conditions=housing_conditions_7, Level_of_disrepair=housing_conditions_8, N_moves_past_year=housing_conditions_9, Currently_receiving_housing_subsidy=housing_conditions_10, Formal_eviction_notice=housing_conditions_11,  Lost_housing_voucher=housing_conditions_12, substance_use_current= Clincial_1, drug_or_alcohol_treatment_current= Clincial_2, Anxiety_disorder= Clincial_8, Emergency_departements_visits= Clincial_11, Depressive_episode_lifetime= Clincial_12, suicidal_ideation_lifetime= Clincial_15, Outpatient_admissions=Clincial_20, Mental_illness= Clincial_21, Chronic_ohysical_health_problem= Clincial_22, Mental_healt_outpatient_service= Clincial_30, conflict_with_landlord = interpersonal_discord_1, conflict_in_household= interpersonal_discord_2, domestic_violence=interpersonal_discord_3, potective_service_involvement=interpersonal_discord_4, discord_rating=interpersonal_discord_5, legal_involvement=interpersonal_discord_7, criminal_justice_involvement=criminal_history_3, childhood_adversity=childhood_experiences_1, foster_care=childhood_experiences_2, childhood_homelessness=childhood_experiences_3, welfare_during_childhood=childhood_experiences_4, childhood_neglet_or_abuse=childhood_experiences_5, Adolescent_mother=childhood_experiences_6, child_food_insecurity_rate= Geographical_4)

#select top 20
row_values <- as.numeric(sum_row[1, ])
sort_sum_rows <- sum_row[order()]
ordered_indices <- order(-row_values)
top_20_cols <- sum_row[, ordered_indices[1:20]]


#transpose for ggplot
sum_row_long <- pivot_longer(top_20_cols, cols = everything(), names_to = "Column", values_to = "Sum")


#add type
type_top_10 <- c("human capital", "demographics", "homeless_history", "homeless_history", "clinical", "clinical", "criminal_history", "demographics", "human_capital", "housing conditions", "demographics:race", "demographics:race", "human capital", "housing conditions", "housing conditions", "clinical", "demographics:race", "human capital", "demographic", "human capital")

#bind, this corresponds to sheet 4.0.
top_20 <- rbind(top_20_cols, type_top_10)
```

```
#transpose top20 in excel, clean names, then import again
top_20_long <- read_excel("/home/kun/Documents/R/Masterarbeit/Appendix/Appendix_B.xlsx", sheet=5)
```

```
#again define colors for ggplot
color_palette <- brewer.pal(n = 8, name = "Dark2")

# plot
Figure_3 <- ggplot(top_20_long, aes(x = reorder(predictor, count), y = count, fill = predictor_type)) +  
  geom_bar(stat = "identity") +
  labs(title = "", x = "", y = "Number of models") +
  theme_classic() +
  theme(
    axis.text.y = element_text(angle = 0, hjust = 1, vjust = 0.5),
    strip.text.y = element_text(angle = 0)  # Ensure facet labels are horizontal
  ) +   guides(fill=FALSE)+
  scale_y_continuous(labels = scales::comma) +  # Format y-axis labels
  facet_grid(predictor_type ~ ., scales = "free_y", space = "free") +  # Use facet_grid with free space and y scales
  scale_fill_brewer(palette = "Dark2") +  # Use fill colors
  coord_flip()  # Flip coordinates to make the bars horizontal


Figure_3
```

# Supplemental Figure 1: Quality measures

Heatmap

```
performance_matrix <- read_excel("/home/kun/Documents/R/Masterarbeit/Appendix/Appendix_B.xlsx", sheet=4)

performance_matrix <- performance_matrix|>
  arrange(Study)

# Melt the data frame for ggplot
data_melted <- melt(performance_matrix, id.vars = "Study")

# Create the heatmap

ggplot(data_melted, aes(x = variable, y = Study, fill = value)) +
  geom_tile(color = "white") +
  scale_fill_manual(values = c("none" = "white", "present" = viridis::viridis(1))) +
  theme_minimal() +
  labs(title = "",
       x = "",
       y = "",
       fill = "Presence") + guides(fill=FALSE)+
  theme(axis.text.x = element_text(angle = 45, hjust = 1))
```

```
sessionInfo()
```

```
R version 4.4.0 (2024-04-24)
Platform: x86_64-pc-linux-gnu
Running under: Ubuntu 24.04.2 LTS

Matrix products: default
BLAS:   /usr/lib/x86_64-linux-gnu/blas/libblas.so.3.12.0 
LAPACK: /usr/lib/x86_64-linux-gnu/lapack/liblapack.so.3.12.0

locale:
 [1] LC_CTYPE=en_US.UTF-8       LC_NUMERIC=C              
 [3] LC_TIME=de_DE.UTF-8        LC_COLLATE=en_US.UTF-8    
 [5] LC_MONETARY=de_DE.UTF-8    LC_MESSAGES=en_US.UTF-8   
 [7] LC_PAPER=de_DE.UTF-8       LC_NAME=C                 
 [9] LC_ADDRESS=C               LC_TELEPHONE=C            
[11] LC_MEASUREMENT=de_DE.UTF-8 LC_IDENTIFICATION=C       

time zone: Europe/Berlin
tzcode source: system (glibc)

attached base packages:
[1] stats     graphics  grDevices utils     datasets  methods   base     

other attached packages:
 [1] ggbreak_0.1.4      ggh4x_0.3.0        reshape2_1.4.4     patchwork_1.2.0   
 [5] RColorBrewer_1.1-3 ggrepel_0.9.5      kableExtra_1.4.0   readxl_1.4.3      
 [9] knitr_1.47         lubridate_1.9.3    forcats_1.0.0      stringr_1.5.1     
[13] dplyr_1.1.4        purrr_1.0.2        readr_2.1.5        tidyr_1.3.1       
[17] tibble_3.2.1       ggplot2_3.5.1      tidyverse_2.0.0   

loaded via a namespace (and not attached):
 [1] gtable_0.3.5       xfun_0.45          htmlwidgets_1.6.4  tzdb_0.4.0        
 [5] vctrs_0.6.5        tools_4.4.0        generics_0.1.3     yulab.utils_0.2.0 
 [9] fansi_1.0.6        highr_0.11         pkgconfig_2.0.3    ggplotify_0.1.2   
[13] lifecycle_1.0.4    farver_2.1.1       compiler_4.4.0     munsell_0.5.1     
[17] ggfun_0.1.8        htmltools_0.5.8.1  yaml_2.3.8         pillar_1.9.0      
[21] viridis_0.6.5      tidyselect_1.2.1   aplot_0.2.5        digest_0.6.35     
[25] stringi_1.8.4      labeling_0.4.3     fastmap_1.1.1      grid_4.4.0        
[29] colorspace_2.1-0   cli_3.6.2          magrittr_2.0.3     utf8_1.2.4        
[33] withr_3.0.0        scales_1.3.0       timechange_0.3.0   rmarkdown_2.26    
[37] gridExtra_2.3      cellranger_1.1.0   hms_1.1.3          evaluate_0.23     
[41] viridisLite_0.4.2  gridGraphics_0.5-1 rlang_1.1.3        Rcpp_1.0.12       
[45] glue_1.7.0         xml2_1.3.6         svglite_2.1.3      rstudioapi_0.16.0 
[49] jsonlite_1.8.8     R6_2.5.1           plyr_1.8.9         systemfonts_1.0.6 
[53] fs_1.6.4
```
